# Periodic Genotype Shifts in Clinically Prevalent *Mycoplasma pneumoniae* Strains in Japan

**DOI:** 10.3389/fcimb.2020.00385

**Published:** 2020-08-06

**Authors:** Tsuyoshi Kenri, Masato Suzuki, Tsuyoshi Sekizuka, Hitomi Ohya, Yoichiro Oda, Tsutomu Yamazaki, Hiroyuki Fujii, Toru Hashimoto, Hiroshi Nakajima, Chihiro Katsukawa, Makoto Kuroda, Keigo Shibayama

**Affiliations:** ^1^Department of Bacteriology II, National Institute of Infectious Diseases, Tokyo, Japan; ^2^Antimicrobial Resistance Research Center, National Institute of Infectious Diseases, Tokyo, Japan; ^3^Pathogen Genomics Center, National Institute of Infectious Diseases, Tokyo, Japan; ^4^Kanagawa Prefectural Institute of Public Health, Kanagawa, Japan; ^5^Chigasaki Municipal Hospital, Kanagawa, Japan; ^6^Wakaba Children's Clinic, Saitama, Japan; ^7^Kurashiki Central Hospital, Okayama, Japan; ^8^Okayama Prefectural Institute for Environmental Science and Public Health, Okayama, Japan; ^9^Osaka Institute of Public Health, Osaka, Japan; ^10^Graduate School of Life and Environmental Sciences, Osaka Prefecture University, Osaka, Japan

**Keywords:** *Mycoplasma pneumoniae*, *p1* gene, genotyping, MLVA, MLST, whole-genome SNP, macrolide resistance, infectious diseases surveillance

## Abstract

Nationwide increases in *Mycoplasma pneumoniae* pneumonia cases in Japan were reported in 2011, 2012, 2015, and 2016. In this study, we isolated 554 *M. pneumoniae* strains in 4 areas in Japan (Kanagawa, Okayama, Osaka, and Saitama) between 2006 and 2019, and performed genotyping analysis. More than 80% of the strains isolated in 2011 and 2012 harbored type 1 *p1* adhesin gene; however, strains harboring type 2 or its variant *p1* gene increased in 2015 and 2016 and dominated after 2017. These findings suggested that a shift in the prevalent genotype of *M. pneumoniae* clinical strains occurred recently in Japan. More than 90% of the type 1 strains isolated after 2010 harbored macrolide-resistance mutations in their 23S rRNA gene, whereas most type 2 lineage strains had no such mutations. Consequently, the increase in type 2 lineage strains in Japan has reduced the macrolide resistance rate of clinical *M. pneumoniae* strains. During this analysis, we also identified *M. pneumoniae* strains carrying a novel variant type 1 *p1* gene, and we classified it as type 1b. We then sequenced the genomes of 81 selected *M. pneumoniae* strains that we collected between 1976 and 2017 in Japan, and compared them with 156 *M. pneumoniae* genomes deposited in public databases to provide insights into the interpretation of *M. pneumoniae* genotyping methods, including *p1* typing, multiple-locus variable-number tandem repeat analysis (MLVA), multi-locus sequence typing (MLST), and typing by 8 single-nucleotide polymorphism markers (SNP-8). As expected, *p1* typing, MLST, and SNP-8 results exhibited good correlation with whole-genome SNP analysis results in terms of phylogenetic relationships; however, MLVA typing results were less comparable to those of the other methods. MLVA may be useful for the discrimination of strains derived from a single outbreak within a limited area; however, is not reliable for classification of strains collected from distantly separated areas at different time points. This study showed the usefulness of genome-based comparison of *M. pneumoniae* for molecular epidemiology. Genome sequencing of more strains will improve our understanding of global propagation routes of this pathogen and evolutionary aspects of *M. pneumoniae* strains.

## Introduction

*Mycoplasma pneumoniae* is a major cause of community-acquired pneumonia worldwide, especially in children (Parrott et al., 2016; Waites et al., 2017). *M. pneumoniae* is a small bacterium that lacks a cell wall and has a genome of approximately 800 kb. Epidemiological studies have revealed that epidemic peaks occur every 3–7 years (Yamazaki and Kenri, 2016). Between 2010 and 2012, large epidemics have been reported globally, including in Asian and European countries (Chalker et al., 2011; Nir-Paz et al., 2012; Yamazaki and Kenri, 2016). In Japan, large outbreaks were reported in 2015 and 2016 (Oishi et al., 2019). As β-lactams are not effective, macrolides are used as first-line drugs for the clinical treatment of *M. pneumoniae* pneumonia (Yamazaki and Kenri, 2016). However, since 2000, macrolide-resistant *M. pneumoniae* (MRMP) strains have spread widely, especially in East-Asian countries (Meyer Sauteur et al., 2016; Pereyre et al., 2016).

Although the pathogenesis is not fully understood, adhesion to and colonization of the human respiratory epithelium by *M. pneumoniae* is thought to be a critical step. The adhesion of *M. pneumoniae* to epithelial cells is mediated mainly by the attachment organelle—a tip structure present at one cell pole (Krause and Balish, 2001; Miyata and Hamaguchi, 2016). At the surface of this organelle, there are dense clusters of adhesin protein complexes (nap structure). The nap is composed of P1, P40, and P90 proteins, which are encoded by genes in the *p1* operon. The *p1* gene encodes P1, whereas P40 and P90 are formed by posttranslational cleavage of the *orf6* gene product (Ruland et al., 1994; Krause, 1998). *p1* and *orf6* exhibit sequence polymorphism, on the basis of which *M. pneumoniae* strains can be classified into 2 groups (type 1 and 2) (Su et al., 1990; Ruland et al., 1994). More recent genome analyses have revealed that there are 2 distinct genetic lineages of *M. pneumoniae* that are discriminated based on genetic background, including single-nucleotide polymorphism (SNP) patterns. The *p1* and *orf6* polymorphisms are related to these two genetic backgrounds of *M. pneumoniae* (Xiao et al., 2015; Diaz et al., 2017).

We previously reported that in Japan, type 1 and 2 lineage *M. pneumoniae* strains dominate alternately in cycles of ~10 years (Sasaki et al., 1996; Kenri et al., 2008). However, the relationship between the 2 lineages and periodic occurrences of pneumonia is unknown. Recent genotyping studies have revealed a correlation between the *p1* gene type and macrolide resistance (MR). Most of the type 1 strains recently isolated in Asian countries were MRMP, whereas type 2 lineage strains were mostly macrolide-susceptible *M. pneumoniae* (MSMP) (Liu et al., 2009; Katsukawa et al., 2019; Zhao et al., 2019a). To improve our understanding of the epidemiological and evolutionary significance of the presence of these 2 lineages of *M. pneumoniae*, it is very important to continue efforts in isolating and genotyping clinical strains over a long period. In this study, we genotyped 554 *M. pneumoniae* strains isolated over the last 14 years in Japan.

It is important to employ effective genotyping methods for molecular epidemiological studies. In addition to standard *p1* typing, various methods for molecular typing of *M. pneumoniae*, including multiple-locus variable-number tandem-repeat analysis (MLVA), multilocus sequence typing (MLST), and typing by 8 SNP markers (SNP-8), have been developed (Degrange et al., 2009; Brown et al., 2015; Touati et al., 2015). Compared to *p1* typing, these typing methods can distinguish more *M. pneumoniae* genotypes and are thought to have higher resolution. However, the biological meanings and relations of the results obtained by these methods are obscure. Further, typing results obtained by the different methods are not readily comparable.

In this study, we sequenced the genomes of 81 *M. pneumoniae* strains isolated in Japan and we compared them with 156 genomes deposited in public databases. We compared the genomes by whole-genome (WG-) SNP analysis and constructed a phylogenetic tree comprising a total 237 *M. pneumoniae* strains. Finally, we evaluated several *M. pneumoniae* genotyping methods and considered their significance in molecular epidemiological and evolutionary studies.

## Materials and Methods

### *M. pneumoniae* Isolates

In total, 554 *M. pneumoniae* isolates were collected in 4 areas of Japan (Kanagawa, Okayama, Osaka, and Saitama prefectures). The isolates are listed in Supplementary Table 1. Throat swabs of patients suspected to have *M. pneumoniae* infection were inoculated in pleuropneumonia-like organism (PPLO) broth or diphasic medium for isolation of *M. pneumoniae* (Katsukawa et al., 2019). In Kanagawa, 231 isolates were obtained between 2006 and 2018 at Kanagawa Prefectural Institute of Public Health. In Okayama, 115 isolates were obtained between 2008 and 2017 at Kurashiki Central Hospital. In Osaka, 65 throat swabs were collected at Kishiwada Tokushukai Hospital between October 2017 and February 2019. From these 65 swabs, 33 isolates were obtained at Osaka Institute of Public Health. In Saitama, 666 throat swabs were collected at Wakaba Children's Clinic between June 2017 and August 2019, and from these, 175 isolates were obtained at the National Institute of Infectious Diseases. The throat swabs collected in Osaka and part of the specimens from Saitama were also subjected to loop-mediated isothermal amplification (LAMP) for the detection of *M. pneumoniae* DNA. The throat swab specimens from Osaka were analyzed by LAMP using the Loopamp Mycoplasma P kit (Eiken Chemical, Tokyo, Japan) according to the manufacturer's instructions. LAMP of specimens from Saitama was performed at BML General Laboratory (Saitama, Japan). The throat swabs were collected and analyzed under the approval of the ethics committees of Kanagawa Prefectural Institute of Public Health and the National Institute of Infectious Diseases.

### *p1* Typing and Detection of MR Mutations

*p1* genotyping of *M. pneumoniae* isolates was performed by PCR-restriction fragment length polymorphism (RFLP) as reported previously (Cousin-Allery et al., 2000). When a novel PCR-RFLP pattern was found, the *p1* gene was sequenced using a previously reported method and primer set (Katsukawa et al., 2019). Supplementary Figure 1 shows simulated PCR-RFLP patterns of known *p1* gene types that were used to compare and judge experimental PCR-RFLP patterns of the isolates. If the PCR-RFLP pattern change was caused by a spontaneous single nucleotide mutation or an AGT tandem-repeat number change (Zhao et al., 2011), the *p1* was not regarded as a new type in this study. If the pattern change was caused by a novel sequence variation in the *p1* that was considered to be derived from the repertoire of the repetitive sequences (RepMPs) outside the *p1* locus of the *M. pneumoniae* genome, the *p1* gene was regarded as the result of DNA recombination(s) between the *p1* and RepMPs and was classified as a new *p1* type (also see Supplementary Figure 2). MR of the *M. pneumoniae* isolates was examined by detecting MR mutations in the 23S rRNA gene, using a previously reported method (Katsukawa et al., 2019).

### Statistical Analysis

Data were analyzed by Fisher's exact test and the chi-square test with a significance level of α = 0.05 (*P* < 0.05), using R software version 3.2.3.

### Genome Sequencing

Eighty-one Japanese *M. pneumoniae* strains were sequenced in this study (Supplementary Table 2). The strains were cultured in 10 mL of PPLO broth, and cells were harvested. Genomic DNA was extracted using a QIAamp DNA Mini Kit (Qiagen, Hilden, Germany). Libraries of 500–900 bp-long genomic DNA inserts were prepared using the Nextera XT DNA Library Prep Kit (Illumina, San Diego, CA, USA). Next-generation sequencing was carried out using Illumina MiSeq, MiniSeq, and HiSeq 2500 platforms. The obtained paired-end reads were assembled *de novo* using CLC Genomics Workbench 11.0.1 (Qiagen).

### *Orf6* Gene Typing

The *orf6* (MPN142) gene was typed in the genome-sequenced strains. In this study, 5 *orf6* types were distinguished (1, 2, 2b, 2c, and 2f). Table 1 shows the reference strains and the GenBank accession nos. of the sequences of these *orf6* types. *Orf6* types were determined by examining the genome sequence data and by amplifying the *orf6* region using the primers ORF6-F (5′-GCGCCAAAACGCTTGAAACA-3′) and OP-R1 (5′-TTGCACTAGGAAGGTAATGT-3′). The amplified *orf6* region was analyzed by sequencing using previously reported primers (Katsukawa et al., 2019).

**Table 1:** Five *orf6* gene types classified and designated in this study.

***orf6* types**	**Reference strains**	**(*p1* type)**	**Accession no**.
1	M129	(1)	M21519
2	FH	(2)	CP010546
2b	KCH-402	(2b)	AP017318
2c	KCH-405	(2c)	AP017319
2f	M282	(2f)	LC390170

*The names of representative strains and GenBank accession nos. are shown*.

### MLVA, MLST, and SNP-8 Typing

MLVA (Degrange et al., 2009), MLST (Brown et al., 2015), and SNP-8 (Touati et al., 2015) types of the genome-sequenced strains were determined by analyzing the respective marker regions in the genome sequence data. If the variable number tandem repeat (VNTR) number for MLVA could not be determined from the draft genomes, the VNTR marker regions were PCR-amplified using the following modified primer sets for MLVA; Mpn1_FS (5′-ATCAGCAACTTCTAATGAAG-3′), Mpn1_RS (5′-TACTAAAACTTTTTGAGCTA-3′), Mpn13_FS (5′-AATAAAATAGGTGAAGGTGA-3′), Mpn13_RS (5′-ATATTCGTTTAAGAGCCAAA-3′), Mpn14_FS (5′-CAAATTAACTCAAACTGTTG-3′), Mpn14_RS (5′-TGTTGAAAGCCTAATTTTCT-3′), Mpn15_FS (5′-CTTTCATTCAAATCACTAAA-3′), Mpn15_RS (5′-CAAGGTGAGCAAATAAGAAT-3′), Mpn16_FS (5′-TGATCCCAACAGTAAACCCT-3′), and Mpn16_RS (5′-TTCAAAGTAAGCAGATGTAC-3′). Using the same primers, the PCR products were sequenced to determine the VNTR number.

### WG-SNP Analysis

WG-SNP analysis was performed using the CSI Phylogeny 1.4 pipeline (https://cge.cbs.dtu.dk/services/CSIPhylogeny/) (Kaas et al., 2014). The genome sequences of the type 1 strain M129-B7 (GenBank accession no. CP003913) and the type 2 strain FH (GenBank accession no. CP017327) were used as reference sequences for analyses of type 1 and type 2 lineage strains, respectively. Phylogenetic trees were visualized using FigTree v1.4.2 software.

### Nucleotide Sequence Accession Numbers

The type 1b *p1* gene sequence of strain KP2440 was deposited into the DDBJ/ENA/GenBank databases under the accession number LC388569. Genome sequence data were deposited under the accession numbers DRA003754, BLFQ01000000, BLFR01000000, BLFS01000000, BLFT01000000, BLFU01000000, BLFV01000000, BLFW01000000, BLFX01000000, BLFY01000000, BLGC01000000, BLGD01000000, BLGE01000000, BLGF01000000, BLGG01000000, BLGH01000000, BLGI01000000, BLGJ01000000, BLGK01000000, BLGL01000000, BLGM01000000, BLGN01000000, BLGO01000000, BLGP01000000, BLGQ01000000, BLGR01000000, BLGS01000000, BLGT01000000, BLFZ01000000, BLGA01000000, BLGB01000000, BLGU01000000, BLGV01000000, BLGW01000000, BLGX01000000, BLGY01000000, BLGZ01000000, BLHA01000000, BLHB01000000, BLHC01000000, BLHD01000000, BLHE01000000, BLHF01000000, BLHG01000000, BLHH01000000, BLHI01000000, BLHJ01000000, BLHK01000000, BLHL01000000, BLHM01000000, BLHN01000000, BLHO01000000, BLHP01000000, BLHQ01000000, BLHR01000000, BLHS01000000, BLHT01000000, BLHU01000000, BLHV01000000, BLHW01000000, BLHX01000000, BLHY01000000, BLHZ01000000, BLIA01000000, BLIB01000000, and BLIC01000000.

## Results

### *M. pneumoniae* Isolates

**Figure 1:**
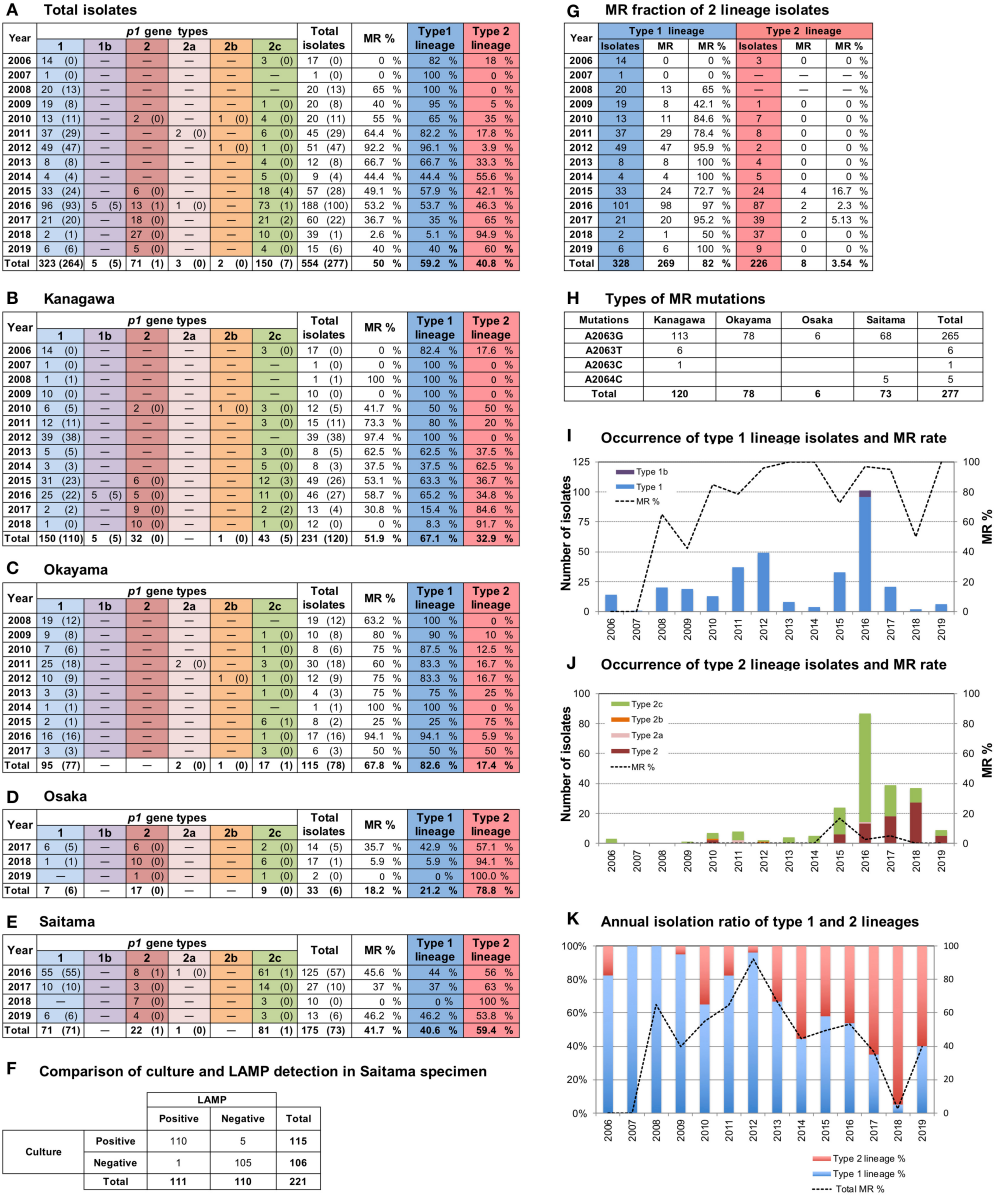
Results of *p1* genotyping and macrolide resistance (MR) detection of the 554 *M. pneumoniae* isolates collected in this study. **(A)** Annual numbers of total *p1* type isolates. Merged data of **(B–E)**. **(B–E)** Annual numbers of *p1* type isolates in the 4 study areas (Kanagawa, Okayama, Osaka, and Saitama) are shown. Numbers of isolates carrying MR mutations are shown in parentheses. **(F)** LAMP and culture test results for the 221 swab specimens collected in Saitama. **(G)** Annual numbers of isolates shown by type 1 and 2 lineages. Fraction and rate of MR of two lineages are shown. **(H)** Numbers and types of MR mutations detected in this study. **(I)** Occurrence of type 1 lineage isolates. Bars indicate annual isolation number of type 1 lineage isolates, the dotted line indicates the MR rate of type1 lineage isolates. **(J)** Occurrence of type 2 lineage isolates. Bars indicate annual isolation number of type 2 lineage isolates; the dotted line indicates the MR rate of type 2 lineage isolates. **(K)** Annual isolation rates of type 1 (blue) and 2 (red) lineages. The dotted line indicates the total MR rate of isolates.

A total of 554 *M. pneumoniae* isolates were collected in 4 areas in Japan between 2006 and 2019 in this study (Figure 1A); 231 were isolated in Kanagawa, 115 in Okayama, 33 in Osaka, and 175 in Saitama (Figures 1B–E). The 33 isolates from Osaka (Figure 1D) were cultured from 65 throat swabs (50.8%), which all tested positive for *M. pneumoniae* DNA by LAMP. The 175 isolates from Saitama (Figure 1E) were obtained from 666 throat swabs, 221 of which were also subjected to the LAMP test. Of these 221 swabs, 110 were *M. pneumoniae*-positive by LAMP test and culture, 1 specimen was LAMP-positive and culture-negative, 5 were LAMP-negative and culture-positive, and the remaining 105 specimens tested negative by both methods (Figure 1F). LAMP was more sensitive than culture detection of *M. pneumoniae* for the Osaka specimens. However, the detection rates by LAMP or culture were similar for Saitama specimens. This was probably due to differences in test procedures and in conditions of specimens between Osaka and Saitama. The collection period and number of *M. pneumoniae* isolates varied widely depending on the study area (Figures 1B–E). Annual isolation numbers were associated with the *M. pneumoniae* pneumonia epidemic patterns. Numerous isolates were obtained in 2011, 2012, 2015, and 2016 (Figures 1I,J), when large outbreaks of *M. pneumoniae* pneumonia occurred in Japan (**Figure 3B**). Detailed surveillance data of *M. pneumoniae* pneumonia in Japan are shown in Supplementary Figure 3.

### *p1* Typing of the *M. pneumoniae* Isolates

**Figure 2:**
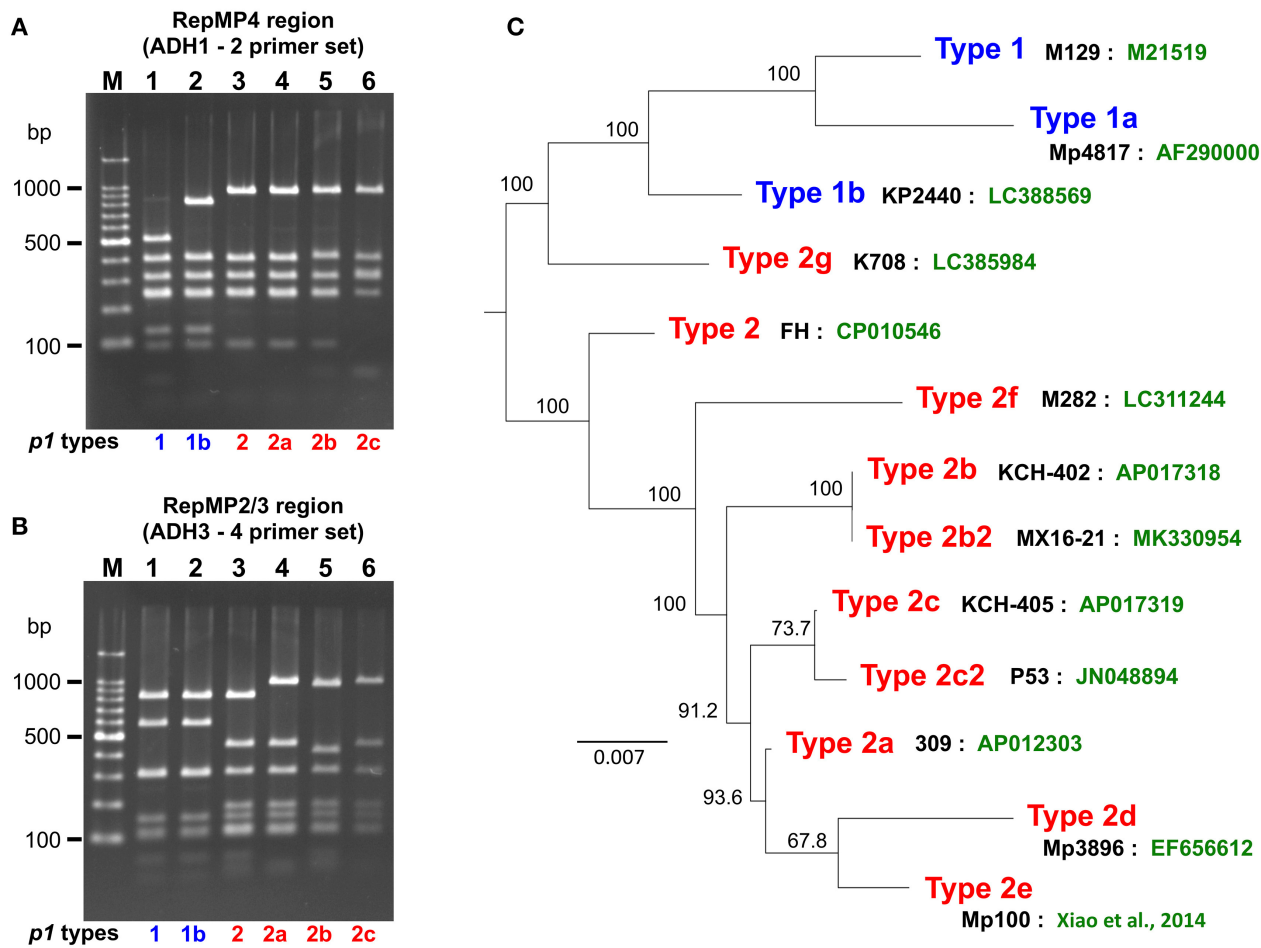
PCR-RFLP analysis of the new type *p1* gene. **(A)** RFLP patterns of the RepMP4 region obtained using the ADH1-2 primer set. **(B)** RFLP patterns of the RepMP2/3 region obtained using ADH3-4 primer set. PCR products were digested with *Hae*III and analyzed by 2% agarose-gel electrophoresis. Lane M: 100-bp ladder marker, lane 1: M129 (type 1), lane 2: KP2440 (type 1b), lane 3: FH (type 2), lane 4: 309 (type 2a), lane 5: KCH-402 (type 2b), and lane 6: KCH-405 (type 2c). **(C)** Dendrogram of 13 *p1* gene sequences reported to date. The tree was generated by alignment of *p1* gene sequences using ClustalW v.2.1. The names of representative strains of each type (black) and GenBank accession nos. of the sequences (green) are shown. Type 2b is also reported as type 2V (Diaz et al., 2017). Type 2b2 is also reported as type 2e or type 2bv (Alishlash et al., 2019; Gullsby et al., 2019). Type 2e of strain Mp100 was originally reported as type 2d (Xiao et al., 2014). In this study, the *p1* types were classified as shown in this figure for systematic classification.

*p1* genotyping of the 554 *M. pneumoniae* isolates by PCR-RFLP analysis revealed that 323 isolates were type 1, 71 were type 2, 3 were type 2a, 2 were type 2b, 150 were type 2c, and 5 were of a novel *p1* type (Figure 1A). The 5 isolates harboring the novel *p1* type were detected in Kanagawa in 2016 (Figure 1B) and exhibited an identical PCR-RFLP pattern. The patterns for one of the strains harboring the new variant, KP2440, are shown in Figures 2A,B. KP2440 exhibited a novel RFLP pattern in the RepMP4 region analyzed using the ADH1-2 primer set (Figure 2A, lane 2), whereas the pattern of the RepMP2/3 region was identical to that of type 1 *p1* (Figure 2B, lane 2). We sequenced the new *p1* variant and established that it was similar to type 1 *p1* sequence but has a novel variation in RepMP4 region (GenBank accession no. LC388569). Therefore, we concluded that it was created from a type 1 *p1* gene by a recombination in the RepMP4 region, and we designated the new variant type 1b. The variation in RepMP4 region of type 1b was similar to that of type 2d *p1* (GenBank accession no. EF656612), probably because the variation sequence was derived from the same RepMP region (RepMP4-h) (Supplementary Figure 2). Figure 2C shows the dendrogram and accession numbers of the 13 *p1* gene sequences reported to date.

In Kanagawa, 231 isolates were collected between 2006 and 2018. Before 2013, type 1 was the dominant lineage. However, type 2 lineage isolates increased as of 2014, and dominated in 2017 and 2018 (*p* < 0.01; Figure 1B). In Okayama, 115 isolates were collected between 2008 and 2017. Type 1 isolates were dominant before 2014, whereas the type 2 lineage rate increased as of 2015 (*p* < 0.01; Figure 1C). The 33 isolates from Osaka were collected between October 2017 and February 2019. Of these, 26 (79%) were of type 2 lineage (Figure 1D). The 175 isolates from Saitama were collected between June 2016 and August 2019. Approximately 60% of the isolates were of type 2 lineage (Figure 1E). From the integrated data of the *M. pneumoniae* isolates from the 4 areas shown in Figures 1A,K, it appears that clinically prevalent *M. pneumoniae* changed from the type 1 to the type 2 lineage in Japan in the past 10 years.

### MR of the Isolates

Of the 554 isolates, 277 (50%) were MRMP strains harboring a MR point mutation in the 23S rRNA gene (Figure 1A). A2063G was the major MR mutation (265/277, 95.7%). Other MR mutations detected were A2063T (6/277, 2.2%), A2063C (1/277, 0.4%), and A2064C (5/277, 1.8%) (Figure 1H). A2063T and A2063C mutations were detected in Kanagawa isolates, whereas A2064C was detected in Saitama isolates.

The fraction of MR was very high in the type 1 lineage (269/328, 82%), whereas MRMP type 2 lineage isolates were rare (8/226, 3.5%) (*p* < 0.01) (Figures 1G,I,J). This correlation between MR and the *p1* type was commonly observed in the 4 areas (Figures 1B–E). The type 1 isolates obtained in Kanagawa in 2006, 2007, and 2009 (25 in total) were MSMP without MR mutations (Figure 1B); however, most type 1 isolates collected in the 4 areas during and after 2010 were MRMP (Figures 1B–E,G). The overall fraction of MR decreased after 2013 because of an increase in MSMP isolates of type 2 lineage (Figure 1K).

### Integrated Data of Genotyping Analysis of *M. pneumoniae* in Japan

**Figure 3:**
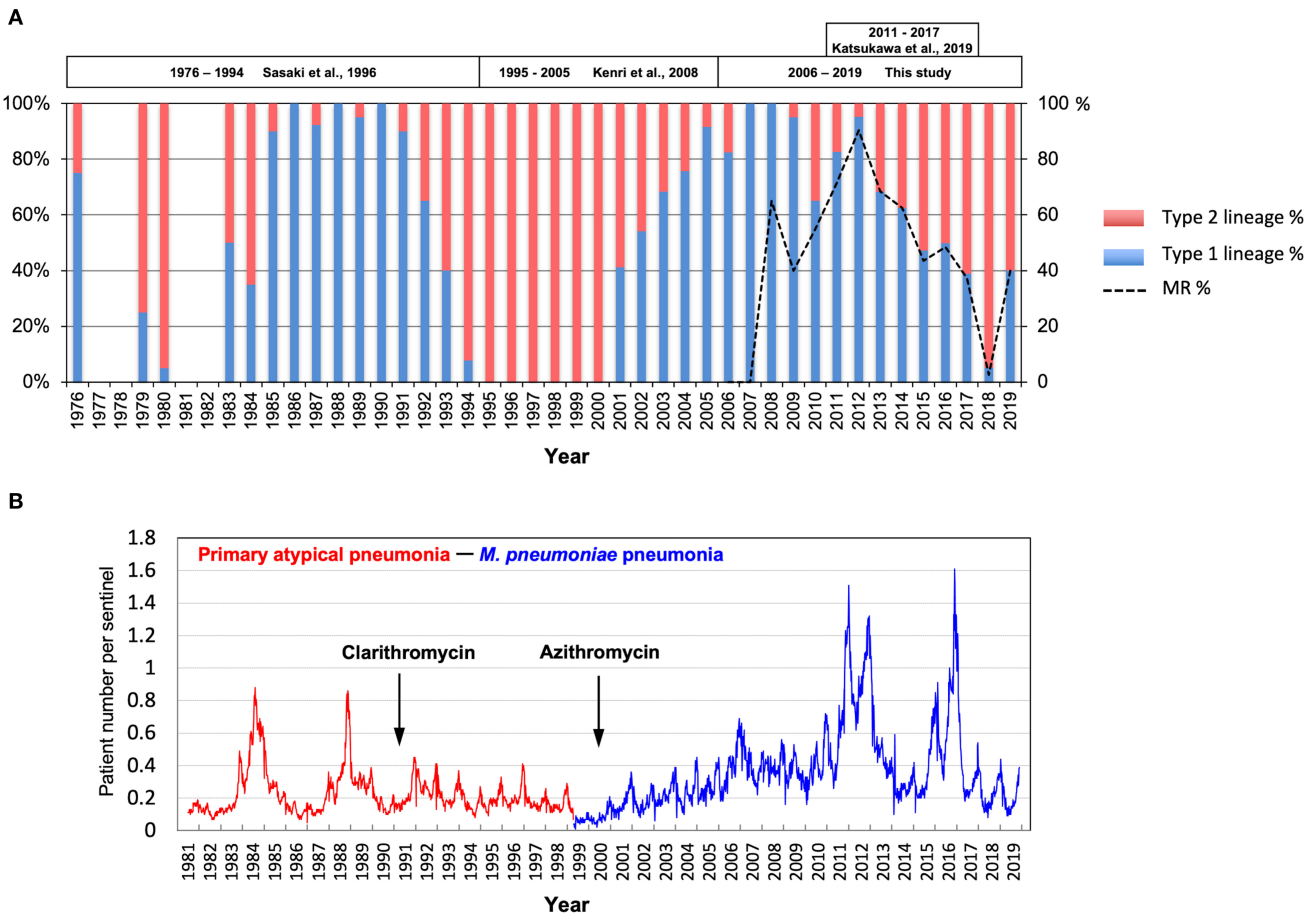
Integrated data of genotyping of *M. pneumoniae* isolates and surveillance of *M. pneumoniae* pneumonia in Japan. **(A)** Integrated data of *p1* gene typing of *M. pneumoniae* isolates over the past 40 years in Japan. Blue and red bars show annual detection rates of type 1 and type 2 lineage strains. The dotted line shows the MR rate of isolates after 2006. Detailed integrated data are shown in Supplementary Figure 4. **(B)** Surveillance data of *M. pneumoniae* pneumonia in Japan by the National Epidemiological Surveillance of Infectious Diseases (NESID) (https://www.niid.go.jp/niid/ja/10/2096-weeklygraph/1659-18myco.html). Surveillance data of primary atypical pneumonia (red line) for April 1981–March 1999, before the start of *M. pneumoniae* pneumonia surveillance (blue line) in April 1999 (Yamazaki and Kenri, 2016) are shown. The years of introduction of clarithromycin and azithromycin for the treatment for *M. pneumoniae* pneumonia in Japan are indicated.

Figure 3A shows integrated *M. pneumoniae* genotyping data from this study and our previous reports (Sasaki et al., 1996; Kenri et al., 2008; Katsukawa et al., 2019). The genotyping data were generated between 1976 and 1994 (isolates in Kanagawa) (Sasaki et al., 1996), between 1995 and 2005 (isolates in Kanagawa, Nagasaki, and Hokkaido) (Kenri et al., 2008), between 2011 and 2017 in Osaka (Katsukawa et al., 2019), and in this study (for details, see Supplementary Figure 4). The latest data of the surveillance of *M. pneumoniae* pneumonia in Japan by the National Epidemiological Surveillance of Infectious Diseases (NESID) are shown in Figure 3B. These data, covering approximately 40 years, revealed that the type 1 and 2 lineage strains dominate alternately, with ~10-year cycles. In the late 1980s and early 1990s, type 1 was dominant, whereas in the late 1990s and the early 2000s, type 2 was dominant. Type 1 became dominant again from the mid-2000s to early 2010s, whereas the type 2 lineage increased again in the latter half of the 2010s. On the other hand, large increases in *M. pneumoniae* pneumonia patients were observed by the NESID in Japan in 1984, 1988, 2011, 2012, 2015, and 2016 (Figure 3B). The relation between these pneumonia epidemic outbreaks and prevalent *M. pneumoniae* type is not apparent. However, most of the isolates in the epidemic periods in 1988, 2011, and 2012 were of type 1, whereas there were a considerable number of type 2 lineage isolates in the epidemic periods in 1984, 2015, and 2016 (Figure 3 and Supplementary Figure 4).

### Genome Sequencing and Phylogenetic Analysis

**Figure 4:**
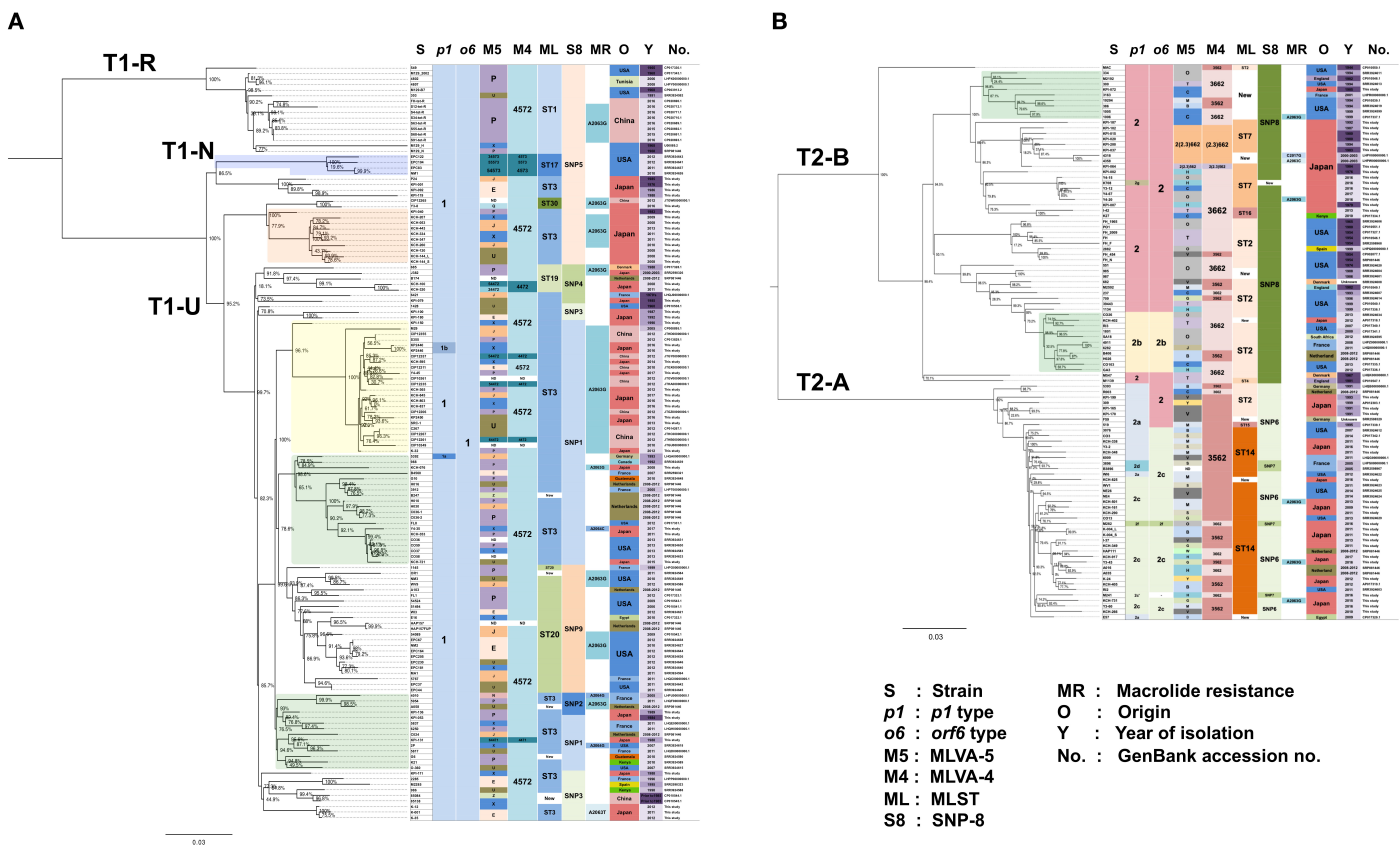
Phylogenetic tree of 237 *M. pneumoniae* genomes based on whole-genome SNP analysis. *p1, orf6*, MLVA, MLST, and SNP-8 types, MR, origin, year of isolation, and GenBank accession nos. of the strains are shown at the right side of the trees. Bootstrap values are indicated at the branches. **(A)** Phylogenetic tree of 136 genome data of *p1* type 1 lineage strains. **(B)** Phylogenetic tree of 101 genomes of *p1* type 2 lineage strains. Magnifications of the trees are shown in Supplementary Figure 5.

To gain a deeper understanding of the genotypes of *M. pneumoniae*, we sequenced the genomes of 81 *M. pneumoniae* isolates that are representatives of our strain collection established between 1976 and 2017 in Japan (Supplementary Table 2). The strains were selected based on the *p1* type, year of isolation, and MR, and included 43 type 1 lineage strains (41 type 1 and 2 type 1b strains) and 38 type 2 lineage strains (15 type 2, 6 type 2a, 15 type 2c, 1 type 2f, and 1 type 2g). We compared the draft genome sequences of these 81 strains with 156 *M. pneumoniae* genomes deposited in public databases (237 strains in total), using WG-SNP analysis. When we compared 237 genomes by using the M129-B7 genome (type 1) as a reference, we detected <300 core-genome SNPs between same *p1* type strains. On the other hand, 750–900 core-genome SNPs were detected when we compared type 1 and 2 lineages. This was also true when we used the FH genome (type 2) as a reference. Thus, the type 1 and 2 lineage strains were completely separated by genome-level comparison as reported previously (Brown et al., 2015; Diaz et al., 2017). Then, we analyzed the type 1 and 2 lineage strains separately by WG-SNP using the reference genomes M129-B7 or FH. Figure 4 shows the phylogenetic trees of type 1 and type 2 lineage strains (for a magnified image, see Supplementary Figure 5).

In line with findings in a previous report (Diaz et al., 2017), the type strain M129 of type 1 lineage was located in a relatively small clade (T1-R), which was separated from a larger clade (T1-U) that contained numerous recent isolates (Figure 4A). None of the 81 isolates sequenced in this study belonged to the T1-R clade. The type 1b strains identified in this study (KP2440 and KP2446) belonged to the T1-U clade (Figure 4A). Type 2 lineage strains were separated into a T2-A clade (containing type 2a and 2c strains) and a T2-B clade (containing type 2 and type 2b strains) (Figure 4B). In these type 2 lineage clades, there were <120 core-genome SNPs between strains of the same clade. However, there were 170–220 core-genome SNPs between different clade strains (T2-A and T2-B).

Strains of a similar origin (similar isolation time or geographic location) tended to cluster together. For example, we observed clusters of strains derived from the USA or Japan, and clusters of old or new strains. MR strains were present in most of the clusters or clades (Figure 4 and Supplementary Figure 5). This suggests that MRMPs were selected independently by macrolide usage from various genetic background populations of *M. pneumoniae*, rather than clonal spread of a single MRMP strain.

For certain strains, multiple genomes were available. For example, for the type strain M129, 4 genomes were available (GenBank accession nos.: CP003913, CP017343, SRP081446, and U00089), and the type 2 type strain FH, 6 (GenBank accession nos.: CP002077, CP010546, CP017327, SRP081446, SRR2598968, and SRR3924606) (Figure 4 and Supplementary Figure 5). The origin of these type strains may be the same; however, their genomes were assigned to slightly different clusters in the WG-SNP phylogenetic tree, probably because of differences in sequencing methods or passage history of these strains in different laboratories. KCH-144_L and KCH-144_S, and K-004_L and K-004_S, which we sequenced in this study, are pairs of strains derived from a same strain (derivatives of a single strain). They were separated during passage cultures on PPLO agar based on colony-size phenotype. They reproductively form large (L) or small (S) colonies on PPLO agar. Genome sequencing data revealed that 2 non-synonymous SNPs between KCH-144_L and KCH-144_S. These non-synonymous SNPs caused a H235N substitution in phenylalanyl-tRNA synthetase subunit alpha (MPN105; *pheS* gene product) and a L56F substitution in sugar ABC transporter ATP-binding protein (MPN258; *yjcW* gene product). Similarly, there was a G101R substitution in phenylalanyl-tRNA synthetase subunit beta (MPN106; *pheT* gene product) between K-004_L and K-004_S. Whether these amino acid substitutions affect the growth rate or colony size of *M. pneumoniae* requires further investigation.

### Evaluation of Genotyping Analysis Methods of *M. pneumoniae*

**Figure 5:**
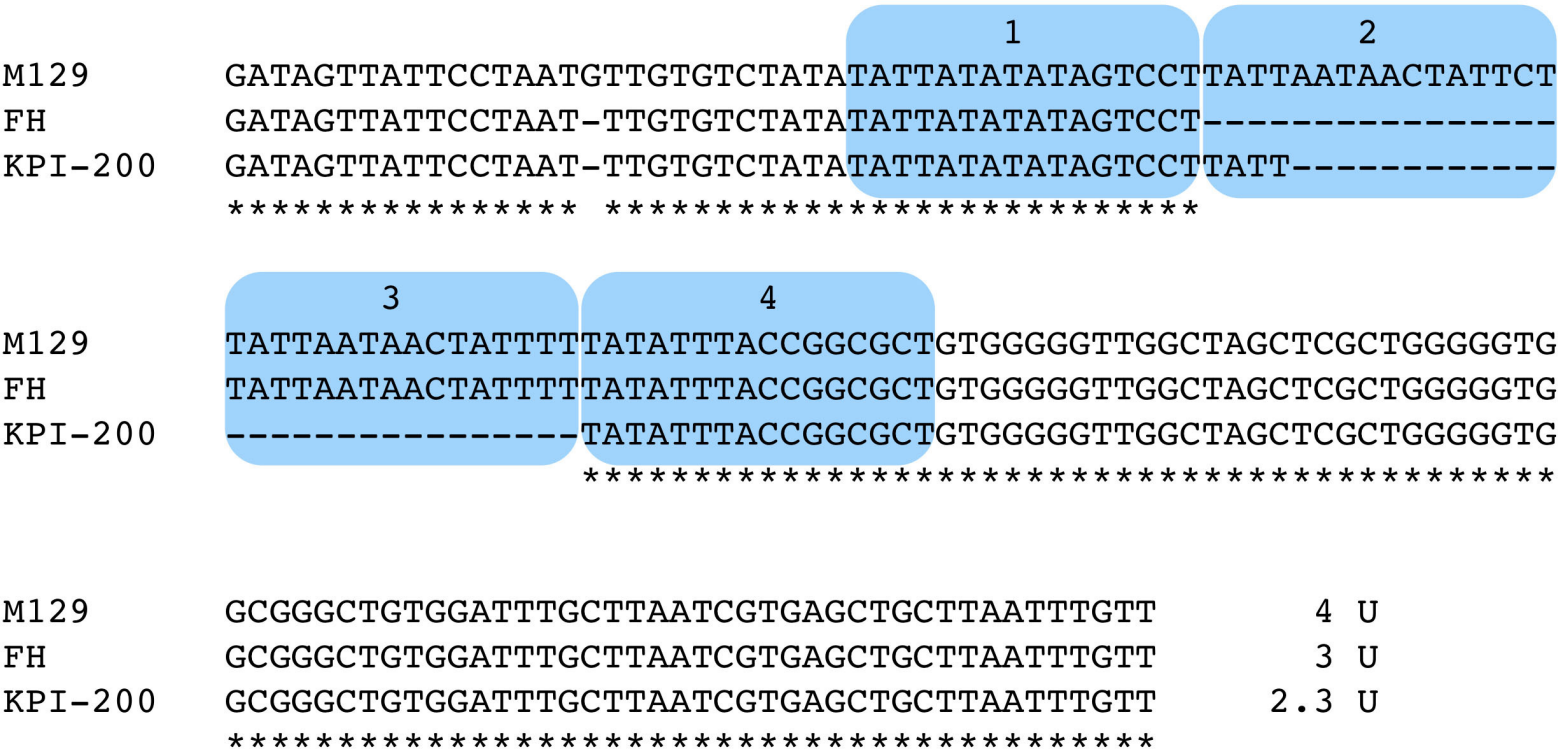
Alignment-based comparison of the Mpn13 VNTR marker region in strains M129, FH, and KPI-200. VNTR repeat units are marked by blue color. The Mpn13 region of strain KPI-200 exhibits irregular repeat number (2.3 units).

To compare and evaluate *M. pneumoniae* genotyping methods, MLVA (Degrange et al., 2009), MLST (Brown et al., 2015), SNP-8 (Touati et al., 2015), and *orf6* (Table 1) types of the 237 genome-sequenced strains were determined (shown at the right side of the phylogenetic trees in Figure 4). Six type 2 strains (KPI-015, KPI-020, KPI-037, KPI-064, KPI-102, and KPI-200) had an irregularly short VNTR marker (Mpn13) in MLVA analysis. These strains were isolated in Kanagawa in the 1980s and 1990s (Supplementary Table 2). Figure 5 shows the nucleotide sequence of the Mpn13 region of these strains. The Mpn13 repeat number in the type strains M129 and FH is 4 and 3, respectively, whereas that in KPI-200, the repeat number was 2.3.

#### MLVA

The MLVA-5 types of the genome-sequenced strains poorly related to their location in the WG-SNP phylogenetic tree (Figure 4 and Supplementary Figure 5). This is mainly due to instability of the Mpn1 marker (Chalker et al., 2015). In MLVA-4, which excludes the Mpn1 marker from MLVA-5, most type 1 strains were classified as the 4572 type. However, the 4 strains in the T1-N clade were of type 4573 (Figure 4A and Supplementary Figure 5). Further analysis is needed to confirm whether Mpn16 = 3 is a common feature of strains that belong to the T1-N clade. Most of the type 2 lineage strains were of 3562 or 3662 type in MLVA-4. The strains belonging to the T2-B clade (type 2 and 2b) tended to be of 3662 type, whereas the strains in the T2-A clade (type 2a and 2c) were mostly 3562 (Figure 4B and Supplementary Figure 5). However, the 3562 type was also found in T2-B and the 3662 type in T2-A.

#### MLST and SNP-8

MLST and SNP-8 typing are based on SNPs between strains. Therefore, the typing results obtained by these methods were relatively comparable to those of WG-SNP analysis. Each MLST and SNP-8 type corresponded to a specific clade or a cluster of the phylogenetic tree. In MLST typing, the type 1 strains of the T1-R clade, including M129, were of type ST1. The strains of the T1-N clade were of type ST17. The strains in T1-U clade were of type ST3, ST19, ST20, or ST30 (Figure 4A and Supplementary Figure 5). The type 2 lineage strains in the T2-B clade were largely ST2, ST7, or ST16. Although most strains in the T2-A clade were ST14, some strains were ST2 (Figure 4B and Supplementary Figure 5). The strains that were ST2 in the T2-A clade were *p1* type 2a strains that harbor type 2 *orf6* (5393, R003, KPI-199, 309, KPI-165, and KPI-170). Other *p1* type 2a strains that had type 2c *orf6* were ST14 (Figure 4B and Supplementary Figure 5).

On the other hand, in the SNP-8 analysis, the SNP5 type was found in the T1-R, T1-N, and part of the T1-U clades (Figure 4A and Supplementary Figure 5). The *p1* type 1 strains that were SNP5 could not be assigned a location in the phylogenetic tree by SNP-8 typing alone. In the type 2 linage, the strains of T2-A and T2-B were largely SNP6 and SNP8, respectively (Figure 4B and Supplementary Figure 5). SNP-8 typing employs a SNP marker that is located in the variable region of *p1* (*p1*^2774^) (Touati et al., 2015). Strains that exhibited SNP7 or new SNP types were *p1* type 2d, 2f, or 2g strains that had a variant *p1*^2774^ marker site, and the M241 strain, which has a large deletion in the *p1* operon (Katsukawa et al., 2019).

## Discussion

We previously reported *p1* gene genotyping studies of *M. pneumoniae* strains isolated in Japan between 1976 and 2005, and showed genotype shifts in clinically prevalent *M. pneumoniae* strains (Sasaki et al., 1996; Kenri et al., 2008; Katsukawa et al., 2019). In this study, we genotyped 554 *M. pneumoniae* isolates collected between 2006 and 2019 in Japan, which revealed that another genotype shift of clinically prevalent strains from type 1 to 2 lineage occurred during the past 14 years (Figure 1K). Moreover, our integrated genotyping data suggested that type shifts between the 2 lineages occurred repeatedly at 10-year intervals in the past 40 years (Figure 3 and Supplementary Figure 4). However, strain numbers in our dataset largely varied, depending on the study area and the year of isolation. A type shift of prevalent *M. pneumoniae* strains has been reported in several other genotyping studies in the other areas of the world; however, repeated type shifts over a long period had not been reported (Pereyre et al., 2007; Dumke et al., 2010; Diaz et al., 2015; Jacobs et al., 2015; Edelstein et al., 2016; Zhao et al., 2019b). To confirm this type shift phenomenon, more extensive and detailed genotyping analyses of *M. pneumoniae* isolates are needed.

Most of the type 1 *p1 M. pneumoniae* strains isolated in recent years in East Asian countries, including China and Japan, were MRMP, whereas MRMP is rare among type 2 lineage strains (Liu et al., 2009; Katsukawa et al., 2019; Zhao et al., 2019a). MRMP strains emerged in and widely spread after 2000 in these countries (Matsuoka et al., 2004; Morozumi et al., 2008; Zhao et al., 2013, 2014; Waites et al., 2017). This is probably because of the extensive use of macrolides for the treatment of *M. pneumoniae* pneumonia in these countries. Initially, MRMP strains were predominantly derived from pediatric patients and rarely detected in adult patients, likely because tetracyclines and quinolones are contraindicated in children younger than 8 years, but are often used for the treatment of adults (Yamazaki and Kenri, 2016). In the 2000s, clinically prevalent *M. pneumoniae* strains were of type 1 lineage and thus, pneumonia is thought to have been caused mainly by type 1 strains in those years (Figure 4A). Pediatric patients were mainly treated with macrolides and therefore, MRMP strains of type 1 would have selectively increased. Type 2 lineage strains were also detected in this period, but at low frequencies, and they therefore did not frequently acquire resistance and largely remained MSMP. Probably, there would be no apparent difference in the frequency of MR acquisition between type 1 and 2 lineages when they are under similar condition and selection pressure by macrolides. Selective increase of type 1 MRMP is not because of the difference in genetic background between type 1 and 2 lineages; it was the result of the prevalence of type 1 strains in pneumonia cases in this period. Furthermore, the onset of pneumonia by type 2 lineage increased in later 2010s. This genotype-shift phenomenon may be caused by immunological status change in the human population, i.e., a stronger protective immunity against type 1 during the type 1 prevalent period may be induced. This genotype-shift phenomenon could occur independently of clinical macrolide usage. Therefore, at the early stage of the genotype-shift phenomenon from type 1 to 2, the type 2 lineage strains remained MSMP. This is a hypothesis that needs to be discussed and investigated. Nevertheless, this scenario well explains the genotyping results in this study and the recent decrease in the MR detection rate of *M. pneumoniae* isolates in Japan. If this scenario is true, it is circumstantial evidence of the actual occurrence of the genotype-shift phenomenon. If the type 2 lineage continues to dominate for several years after this, there is a possibility that MRMP will increase among type 2 lineage strains (Zhao et al., 2019b). Thus, monitoring of the genotype and MR rate of *M. pneumoniae* isolates and evaluation of the relationships with the clinical use of macrolides are particularly important.

Periodic *M. pneumoniae* pneumonia outbreaks have been reported in numerous epidemiological studies worldwide (Foy et al., 1979; Lind et al., 1997; Chalker et al., 2011; Blystad et al., 2012; Kim et al., 2015). This periodicity is probably due to interactions between *M. pneumoniae* and the immunological status of the human population, and is one of the characteristic features of this disease (Yamazaki and Kenri, 2016). In the NESID surveillance in Japan, large increases in pneumonia patients were observed in 1984 and 1988 and that were thought to be periodic epidemic outbreaks (Figure 3B). Although such outbreaks were not apparently observed during the 1990s and 2000s, periodic increases in patients appeared again in 2011, 2012, 2015, and 2016. The reasons for the absence of periodic patient increases in the 1990s and 2000s are unknown; however, it is possible that clarithromycin and azithromycin, which were used for treatment of *M. pneumoniae* pneumonia in these periods, were very effective, and prevented large epidemics (Figure 3B). MRMP strains spread in the 2000s and further into the early 2010s (Figure 3A) and therefore, clarithromycin and azithromycin would have no longer been effective in controlling the disease and thus, periodic epidemic outbreaks (intrinsic feature of this disease) could occur again in 2011 and 2012 (Figure 3A and Supplementary Figure 4). There are no apparent correlations between the *p1* type shift patterns and the epidemic outbreaks observed by the NESID. However, MSMP type 2 lineage strains increased in the 2015 and 2016 outbreaks and dominate at present. In this situation, periodic epidemic outbreaks might disappear again because macrolides are clinically effective again in controlling pneumonia epidemic outbreaks.

Reliable and effective genotyping methods are key for detailed epidemiological and evolutionary studies of bacterial pathogens. MLVA, MLST, and SNP-8 have been used for *M. pneumoniae* in addition to *p1* typing. Besides *p1* typing, MLVA is the most common and widely used method. In MLVA-5 of *M. pneumoniae*, 5 VNTR markers, i.e., Mpn1, Mpn13, Mpn14, Mpn15, and Mpn16, are employed (Degrange et al., 2009). However, it is known that Mpn1 is unstable, and it has been recommended to remove from the analysis and use MLVA-4 for genotyping (Chalker et al., 2015). Compared to MLVA-5, the number of genotypes is greatly decreased in MLVA-4. In this study, most of the type 1 strains were 4572, whereas type 2 lineage strains were mostly 3562 or 3662 (Figure 4). This is because the Mpn13 and Mpn15 markers are well-conserved in the type 1 and 2 lineages. In the type 1 lineage, most strains have “Mpn13 = 4 and Mpn15 = 7,” whereas most type 2 strains exhibit “Mpn13 = 3 and Mpn15 = 6” (Zhao et al., 2019a). However, in this study, several type 2 strains were “Mpn13 = 2.3” (Figures 4B, 5), and the 4 type 1 strains in the T1-N clade were 4573 due to “Mpn16 = 3” (4A, light blue square). None of the genome-sequenced strains in this study belonged to the T1-N clade. If new strains of the T1-N clade are identified in future studies, it would be interesting to see whether they exhibit “Mpn16 = 3” in MLVA and ST17 in MLST (Figure 4A and Supplementary Figure 5).

We found the same MLVA types even for strains that had considerably different genetic backgrounds in WG-SNP phylogenetic analysis, indicating that strains with the same MLVA type are not necessarily identical or having a similar genetic background. MLVA may be useful for the identification or comparison of strains that were collected from relatively limited area and over a short period, e.g., strains collected from a sporadic outbreak in a school or hospital. However, the MLVA cannot be used for the comparison or phylogenetic analysis of strains that are collected from a wide area over a long period. On the other hand, when MLST and SNP-8 types are the same, strains are considered to have a similar genetic background and may be phylogenetically related. However, in this study, certain MLST and SNP-8 types could not be assigned a location in the phylogenetic tree (e.g., ST2 of MLST or SNP5 of SNP-8); in these cases, the MLST or SNP-8 type alone was not sufficient. The use of both typing methods or in combination with *p1* typing may improve the identification and classification of these strains.

The major aim of the phylogenetic analysis of *M. pneumoniae* strains by WG-SNP analysis in this study was to evaluate and compare the genotyping methods (*p1* typing, MLVA, MLST, and SNP-8). The strains used for the analysis had various origins and were not deemed suitable for tracking of the strains to investigate geographical routes of propagation. However, some molecular epidemiological information can be obtained from the WG-SNP phylogenetic tree. For example, a number of strains isolated in Okayama between 2008 and 2013 (Figure 4A and Supplementary Figure 5, light orange square on the tree) are similar to the KPI-040 strain isolated in Kanagawa in 1983 (Supplementary Table 2). The cluster of MRMP strains isolated in Japan and China between 2012 and 2017 (Figure 4A and Supplementary Figure 5, light yellow square on the tree) is related to KPI-150 (MSMP) isolated in Japan in 1990 and M29 (MRMP) isolated in China in 2005. There are also clusters of strains isolated in distantly separated countries (Figure 4 and Supplementary Figure 5, light green squares on the tree). The availability of more genome sequence data of additional strains may increase the resolution of these clusters and might enable the tracking of global propagation routes of *M. pneumoniae*. From the phylogenetic tree of type 2 lineage strains, evolutional aspects of *M. pneumoniae* strains can be speculated upon. When the T2-A and T2-B clades branched, the first type 2a strain might have been a type 2a *p1* and type 2 *orf6* strain in the T2-A clade, such as R003, KPI-199, or 309. Then, RepMP5 recombination of *orf6* occurred in these type 2a strains, generating strains that have type 2a *p1* and type 2c *orf6*, such as CO3, KCH-338, and Y3-2. Later, RepMP4 recombination of *p1* occurred in these strains, generating type 2c strains that carry type 2c *p1* and type 2c *orf6* (Figure 4B and Supplementary Figure 5). These type 2c strains likely spread widely and at present, are frequently isolated from patients with pneumonia.

Thus, phylogenetic analysis based on WG-SNPs may provide useful information on molecular epidemiology and improve our understanding of evolutional aspects of *M. pneumoniae* strains. As *M. pneumoniae* has a small genome, it is relatively easy to obtain genome data of a large number of strains. In future studies, genome analyses of more strains will provide information about features such as genomic SNP accumulation time, global propagation routes, and evolution of *M. pneumoniae* strains. If the type-shift phenomenon of *M. pneumoniae* really occurs, it is probable that there are some antigenic differences between type 1 and 2 lineage strains (Yamazaki and Kenri, 2016). To identify these antigenic differences, genome-level comparative analysis of many *M. pneumoniae* strains will be useful.

## Data Availability Statement

The datasets generated for this study can be found in the DDBJ/ENA/GenBank databases, with accession numbers LC388569, DRA003754, BLFQ01000000, BLFR01000000, BLFS01000000, BLFT01000000, BLFU01000000, BLFV01000000, BLFW01000000, BLFX01000000, BLFY01000000, BLGC01000000, BLGD01000000, BLGE01000000, BLGF01000000, BLGG01000000, BLGH01000000, BLGI01000000, BLGJ01000000, BLGK01000000, BLGL01000000, BLGM01000000, BLGN01000000, BLGO01000000, BLGP01000000, BLGQ01000000, BLGR01000000, BLGS01000000, BLGT01000000, BLFZ01000000, BLGA01000000, BLGB01000000, BLGU01000000, BLGV01000000, BLGW01000000, BLGX01000000, BLGY01000000, BLGZ01000000, BLHA01000000, BLHB01000000, BLHC01000000, BLHD01000000, BLHE01000000, BLHF01000000, BLHG01000000, BLHH01000000, BLHI01000000, BLHJ01000000, BLHK01000000, BLHL01000000, BLHM01000000, BLHN01000000, BLHO01000000, BLHP01000000, BLHQ01000000, BLHR01000000, BLHS01000000, BLHT01000000, BLHU01000000, BLHV01000000, BLHW01000000, BLHX01000000, BLHY01000000, BLHZ01000000, BLIA01000000, BLIB01000000, and BLIC01000000.

## Ethics Statement

Clinical isolates were collected and analyzed under the approval of the ethics committees of Kanagawa Prefectural Institute of Public Health and the National Institute of Infectious Diseases.

## Author Contributions

TK, CK, TY, and KS designed the research. HO, YO, TY, HF, TH, HN, and CK organized specimen collection. TK, CK, HO, MS, TS, and HF performed the experiments. TK, MS, TS, and MK analyzed the data. TK wrote the draft manuscript. All authors helped writing and revised the manuscript, reviewed the manuscript, and approved the final version.

## Conflict of Interest

The authors declare that the research was conducted in the absence of any commercial or financial relationships that could be construed as a potential conflict of interest.
