# Supplementary material for: Periodic Genotype Shifts in Clinically Prevalent Mycoplasma pneumoniae Strains in Japan
Source: Front Cell Infect Microbiol. 2020 Aug 6;10:385. doi: 10.3389/fcimb.2020.00385 (PMC7424021; doi:10.3389/fcimb.2020.00385)
Supplement: Supplementary file 1 [file Data_Sheet_1.zip › Figure S1.pdf]

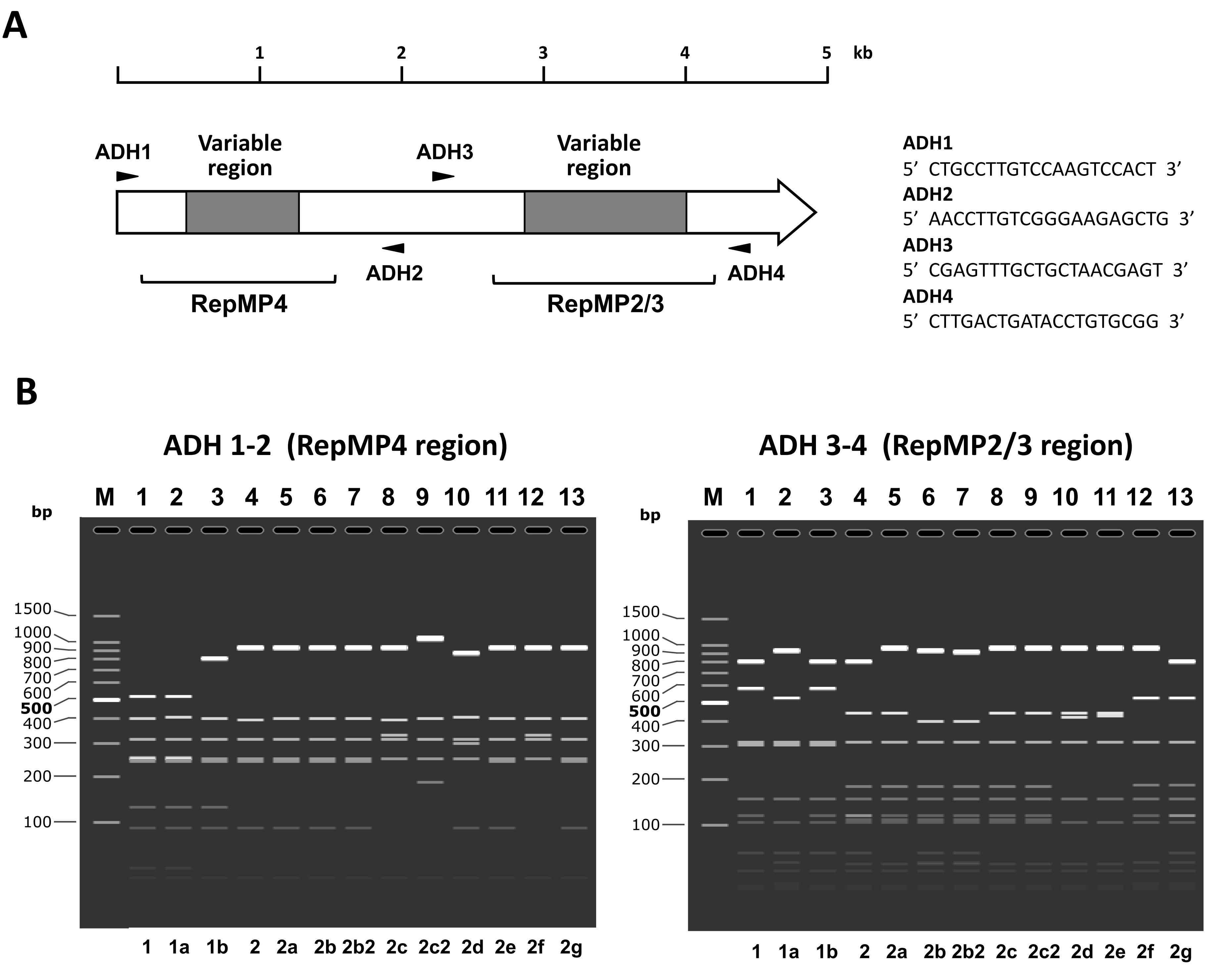

M : Size marker : 100 bp ladder

1 :	M129	(1)	M21519	6 :	KCH-402	(2b)	AP017318	11 :	Mp100	(2e)	(Xiao et al., 2014)
2 :	Mp4817	(1a)	AF290000	7 :	MX16-21	(2b2)	MK330954	12 :	M282	(2f)	LC311244
3 :	KP2440	(1b)	LC388569	8 :	KCH-405	(2c)	AP017319	13 :	K708	(2g)	LC385984
4 :	FH	(2)	CP010546	9 :	p53	(2c2)	JN048894				
5 :	309	(2a)	AP012303	10 :	Mp3896	(2d)	EF656612				

Supplementary Figure S1

Simulation of the PCR-RFLP patterns of 13 *p1* genes reported to date. **(A)** Schematic illustration of the *p1* gene. Approximate positions of PCR primers (ADH1, ADH2, ADH3, and ADH4), sequence variable regions, and repetitive regions (RepMP4 and repMP2/3) are indicated. ADH primer sequences are shown on the right. **(B)** Simulation of PCR-RFLP patterns based on nucleotide sequences of 13 types of *p1* genes (GenBank accession nos. and strain names are shown at the bottom). 2% agarose-gel electrophoresis patterns of ADH1-2 and ADH3-4 PCR products after digestion with *Hae*III were simulated using SnapGene software version 4.3.10 (Snapgene.com).

Xiao, J., Liu, Y., Wang, M., Jiang, C., You, X., and Zhu, C. (2014). Detection of *Mycoplasma pneumoniae* P1 subtype variations by denaturing gradient gel electrophoresis. Diagn Microbiol Infect Dis 78, 24-28.
