# Supplementary material for: Periodic Genotype Shifts in Clinically Prevalent Mycoplasma pneumoniae Strains in Japan
Source: Front Cell Infect Microbiol. 2020 Aug 6;10:385. doi: 10.3389/fcimb.2020.00385 (PMC7424021; doi:10.3389/fcimb.2020.00385)
Supplement: Supplementary file 1 [file Data_Sheet_1.zip › Figure S2.pdf]

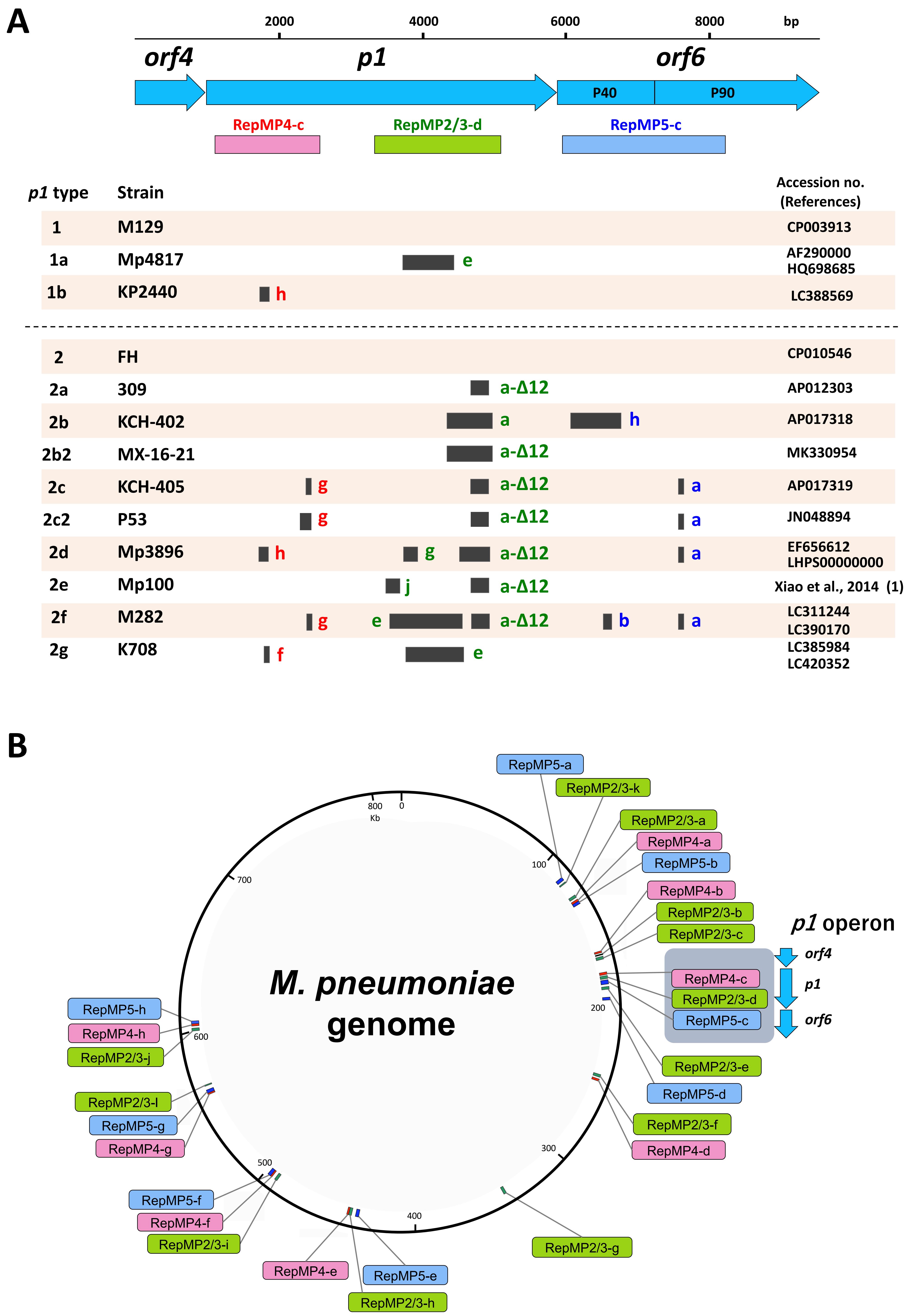

Supplementary Figure S2

Comparison and classification of *p1* and *orf6* gene subtypes of *M. pneumoniae*. **(A)** Schematic illustration of the *p1* operon and comparison of the 13 *p1* gene types. The 3 blue arrows indicate *orf4* (MPN140), *p1* (MPN141), and *orf6* (MPN142) genes of the *p1* operon. The approximate size of the operon (bp) is shown above. Positions corresponding to P40 and P90 proteins are shown in the *orf6* gene. Pink, green, and blue rectangles indicate approximate positions of RepMPs in *p1* (RepMP4-c and RepMP2/3-d) and *orf6* (RepMP5-c). Gray rectangles indicate approximate positions of sequence variation sites in *p1* subtypes. Red, green, and blue characters indicate the suffixes of RepMPs shown in panel **B**. Recombination(s) between these RepMPs and the *p1* operon probably generated these variations. Names of strains that harbor the subtype genes are indicated on the left. Accession numbers of the subtype sequences are shown on the right. Sequence information of *orf6* of type 2b2 and 2e strains have not yet been reported. **(B)** Distribution of RepMPs in the *M. pneumoniae* genome. Approximate positions of 8 RepMP4, 12 RepMP2/3, and 8 RepMP5 regions in the genome are indicated by colored boxes. Suffixes of RepMP regions (-a to -l) are based on the nomenclature system proposed by Spuesens et al. 2009, 2011 (3, 4). Figure adapted from Katsukawa et al. 2019 (4), with some modifications.

1: Xiao, J., Liu, Y., Wang, M., Jiang, C., You, X., and Zhu, C. (2014). Detection of *Mycoplasma pneumoniae* P1 subtype variations by denaturing gradient gel electrophoresis. Diagn Microbiol Infect Dis 78, 24-28.

2: Spuesens, E.B., Oduber, M., Hoogenboezem, T., Sluijter, M., Hartwig, N.G., van Rossum, A.M., and Vink, C. (2009). Sequence variations in RepMP2/3 and RepMP4 elements reveal intragenomic homologous DNA recombination events in *Mycoplasma pneumoniae*. Microbiology 155, 2182-2196.

3: Spuesens, E.B., van de Kreeke, N., Estevao, S., Hoogenboezem, T., Sluijter, M., Hartwig, N.G., van Rossum, A.M., and Vink, C. (2011). Variation in a surface-exposed region of the *Mycoplasma pneumoniae* P40 protein as a consequence of homologous DNA recombination between RepMP5 elements. Microbiology 157, 473-483

4: Katsukawa, C., Kenri, T., Shibayama, K., and Takahashi, K. (2019). Genetic characterization of *Mycoplasma pneumoniae* isolated in Osaka between 2011 and 2017: Decreased detection rate of macrolide-resistance and increase of *p1* gene type 2 lineage strains. PLoS One 14, e0209938.
