# Supplementary material for: Periodic Genotype Shifts in Clinically Prevalent Mycoplasma pneumoniae Strains in Japan
Source: Front Cell Infect Microbiol. 2020 Aug 6;10:385. doi: 10.3389/fcimb.2020.00385 (PMC7424021; doi:10.3389/fcimb.2020.00385)
Supplement: Supplementary file 1 [file Data_Sheet_1.zip › Figure S3.PDF]

A

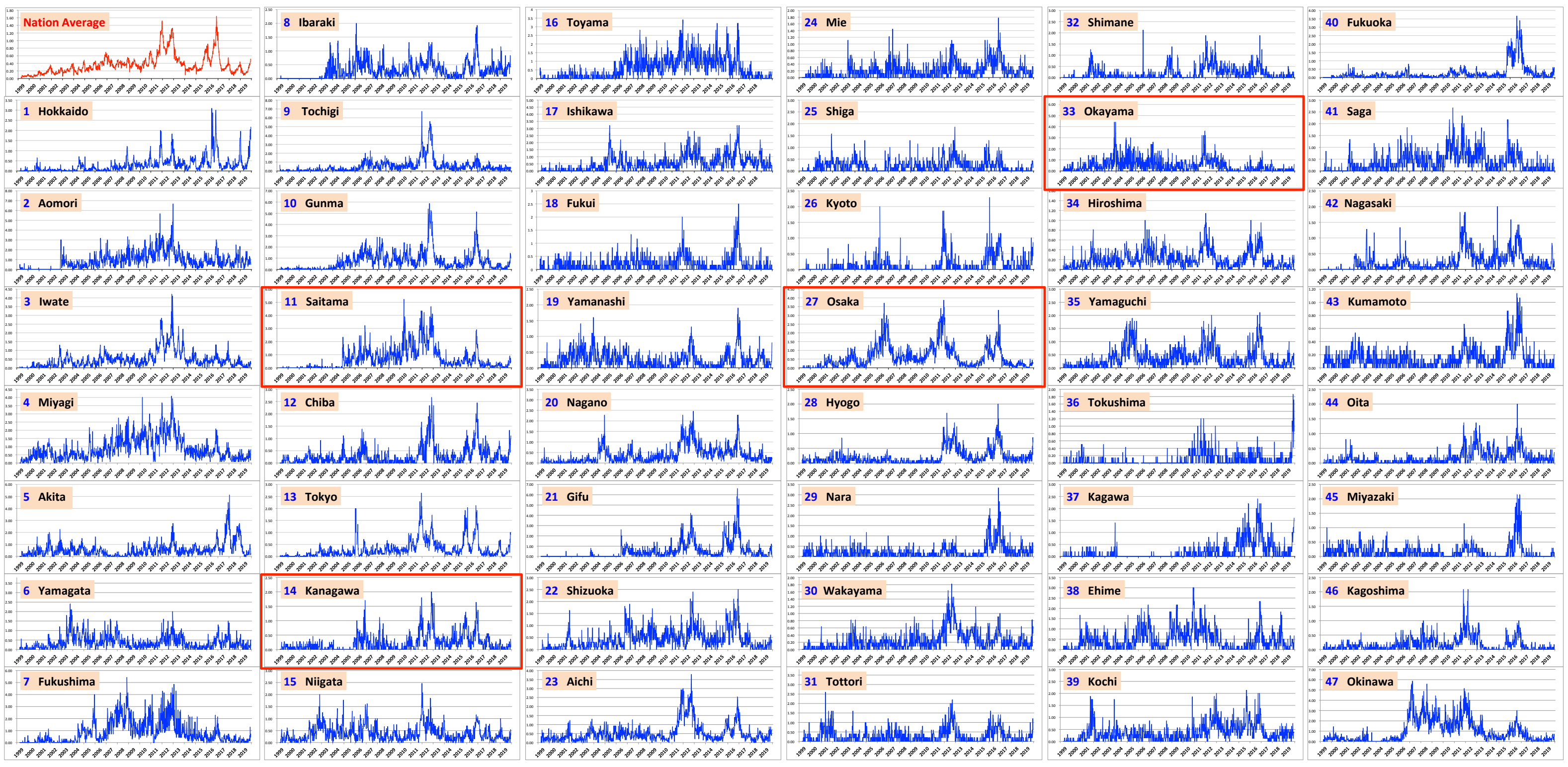

Weekly surveillance data of *Mycoplasma pneumoniae* pneumonia by the prefectures of Japan (1999-2019).

B

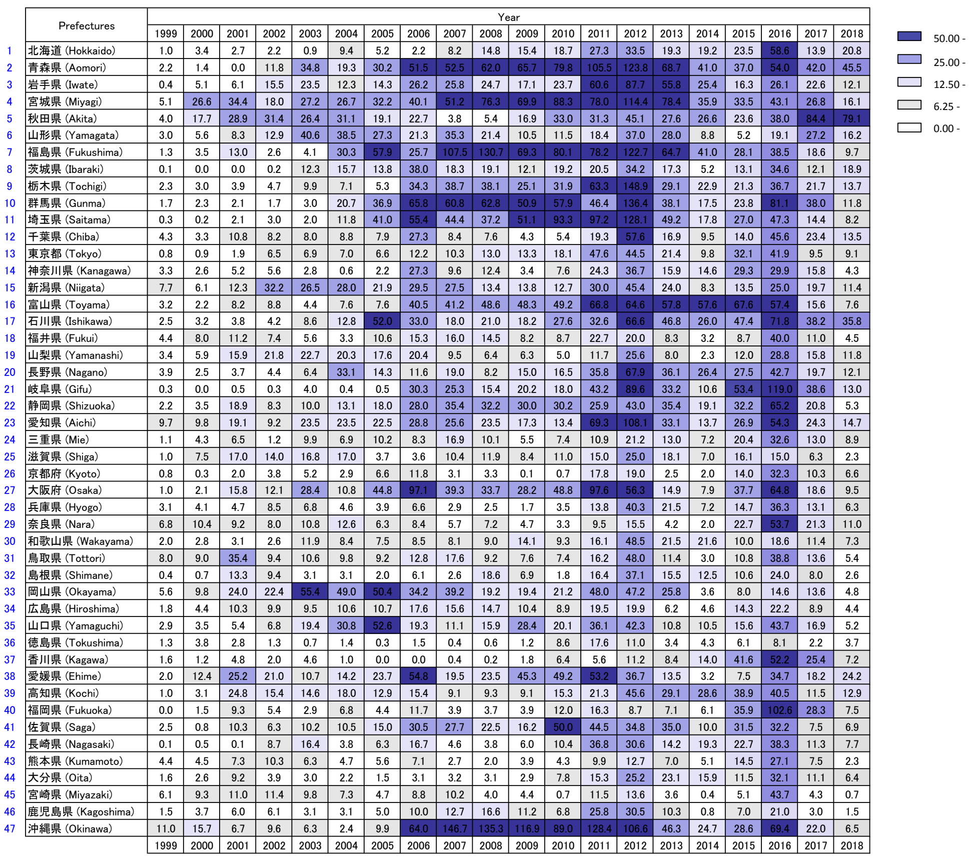

	Prefectures	Year																			
		1999	2000	2001	2002	2003	2004	2005	2006	2007	2008	2009	2010	2011	2012	2013	2014	2015	2016	2017	2018
1	北海道 (Hokkaido)	1.0	3.4	2.7	2.2	0.9	9.4	5.2	2.2	8.2	14.8	15.4	18.7	27.3	33.5	19.3	19.2	23.5	58.6	13.9	20.8
2	青森県 (Aomori)	2.2	1.4	0.0	11.8	34.8	19.3	30.2	51.5	52.5	62.0	65.7	79.8	105.5	123.8	68.7	41.0	37.0	54.0	42.0	45.5
3	岩手県 (Iwate)	0.4	5.1	6.1	15.5	23.5	12.3	14.3	26.2	25.8	24.7	17.1	23.7	60.6	87.7	55.8	25.4	16.3	26.1	22.6	12.1
4	宮城県 (Miyagi)	5.1	26.6	34.4	18.0	27.2	26.7	32.2	40.1	51.2	76.3	69.9	88.3	78.0	114.4	78.4	35.9	33.5	43.1	26.8	16.1
5	秋田県 (Akita)	4.0	17.7	28.9	31.4	26.4	31.1	19.1	22.7	3.8	5.4	16.9	33.0	31.3	45.1	27.6	26.6	23.6	38.0	84.4	79.1
6	山形県 (Yamagata)	3.0	5.6	8.3	12.9	40.6	38.5	27.3	21.3	35.3	21.4	10.5	11.5	18.4	37.0	28.0	8.8	5.2	19.1	27.2	16.2
7	福島県 (Fukushima)	1.3	3.5	13.0	2.6	4.1	30.3	57.9	25.7	107.5	130.7	69.3	80.1	78.2	122.7	64.7	41.0	28.1	38.5	18.6	9.7
8	茨城県 (Ibaraki)	0.1	0.0	0.0	0.2	12.3	15.7	13.8	38.0	18.3	19.1	12.1	19.2	20.5	34.2	17.3	5.2	13.1	34.6	12.1	18.9
9	栃木県 (Tochigi)	2.3	3.0	3.9	4.7	9.9	7.1	5.3	34.3	38.7	38.1	25.1	31.9	63.3	148.9	29.1	22.9	21.3	36.7	21.7	13.7
10	群馬県 (Gunma)	1.7	2.3	2.1	1.7	3.0	20.7	36.9	65.8	60.8	62.8	50.9	57.9	46.4	136.4	38.1	17.5	23.8	81.1	38.0	11.8
11	埼玉県 (Saitama)	0.3	0.2	2.1	3.0	2.0	11.8	41.0	55.4	44.4	37.2	51.1	93.3	97.2	128.1	49.2	17.8	27.0	47.3	14.4	8.2
12	千葉県 (Chiba)	4.3	3.3	10.8	8.2	8.0	8.8	7.9	27.3	8.4	7.6	4.3	5.4	19.3	57.6	16.9	9.5	14.0	45.6	23.4	13.5
13	東京都 (Tokyo)	0.8	0.9	1.9	6.5	6.9	7.0	6.6	12.2	10.3	13.0	13.3	18.1	47.6	44.5	21.4	9.8	32.1	41.9	9.5	9.1
14	神奈川県 (Kanagawa)	3.3	2.6	5.2	5.6	2.8	0.6	2.2	27.3	9.6	12.4	3.4	7.6	24.3	36.7	15.9	14.6	29.3	29.9	15.8	4.3
15	新潟県 (Niigata)	7.7	6.1	12.3	32.2	26.5	28.0	21.9	29.5	27.5	13.4	13.8	12.7	30.0	45.4	24.0	8.3	13.5	25.0	19.7	11.4
16	富山県 (Toyama)	3.2	2.2	8.2	8.8	4.4	7.6	7.6	40.5	41.2	48.6	48.3	49.2	66.8	64.6	57.8	57.6	67.6	57.4	15.6	7.6
17	石川県 (Ishikawa)	2.5	3.2	3.8	4.2	8.6	12.8	52.0	33.0	18.0	21.0	18.2	27.6	32.6	66.6	46.8	26.0	47.4	71.8	38.2	35.8
18	福井県 (Fukui)	4.4	8.0	11.2	7.4	5.6	3.3	10.6	15.3	16.0	14.5	8.2	8.7	22.7	20.0	8.3	3.2	8.7	40.0	11.0	4.5
19	山梨県 (Yamanashi)	3.4	5.9	15.9	21.8	22.7	20.3	17.6	20.4	9.5	6.4	6.3	5.0	11.7	25.6	8.0	2.3	12.0	28.8	15.8	11.8
20	長野県 (Nagano)	3.9	2.5	3.7	4.4	6.4	33.1	14.3	11.6	19.0	8.2	15.0	16.5	35.8	67.9	36.1	26.4	27.5	42.7	19.7	12.1
21	岐阜県 (Gifu)	0.3	0.0	0.5	0.3	4.0	0.4	0.5	30.3	25.3	15.4	20.2	18.0	43.2	89.6	33.2	10.6	53.4	119.0	38.6	13.0
22	静岡県 (Shizuoka)	2.2	3.5	18.9	8.3	10.0	13.1	18.0	28.0	35.4	32.2	30.0	30.2	25.9	43.0	35.4	19.1	32.2	65.2	20.8	5.3
23	愛知県 (Aichi)	9.7	9.8	19.1	9.2	23.5	23.5	22.5	28.8	25.6	23.5	17.3	13.4	69.3	108.1	33.1	13.7	26.9	54.3	24.3	14.7
24	三重県 (Mie)	1.1	4.3	6.5	1.2	9.9	6.9	10.2	8.3	16.9	10.1	5.5	7.4	10.9	21.2	13.0	7.2	20.4	32.6	13.0	8.9
25	滋賀県 (Shiga)	1.0	7.5	17.0	14.0	16.8	17.0	3.7	3.6	10.4	11.9	8.4	11.0	15.0	25.0	18.1	7.0	16.1	15.0	6.3	2.3
26	京都府 (Kyoto)	0.8	0.3	2.0	3.8	5.2	2.9	6.6	11.8	3.1	3.3	0.1	0.7	17.8	19.0	2.5	2.0	14.0	32.3	10.3	6.6
27	大阪府 (Osaka)	1.0	2.1	15.8	12.1	28.4	10.8	44.8	97.1	39.3	33.7	28.2	48.8	97.6	56.3	14.9	7.9	37.7	64.8	18.6	9.5
28	兵庫県 (Hyogo)	3.1	4.1	4.7	8.5	6.8	4.6	3.9	6.6	2.9	2.5	1.7	3.5	13.8	40.3	21.5	7.2	14.7	36.3	13.1	6.3
29	奈良県 (Nara)	6.8	10.4	9.2	8.0	10.8	12.6	6.3	8.4	5.7	7.2	4.7	3.3	9.5	15.5	4.2	2.0	22.7	53.7	21.3	11.0
30	和歌山県 (Wakayama)	2.0	2.8	3.1	2.6	11.9	8.4	7.5	8.5	8.1	9.0	14.1	9.3	16.1	48.5	21.5	21.6	10.0	18.6	11.4	7.3
31	鳥取県 (Tottori)	8.0	9.0	35.4	9.4	10.6	9.8	9.2	12.8	17.6	9.2	7.6	7.4	16.2	48.0	11.4	3.0	10.8	38.8	13.6	5.4
32	島根県 (Shimane)	0.4	0.7	13.3	9.4	3.1	3.1	2.0	6.1	2.6	18.6	6.9	1.8	16.4	37.1	15.5	12.5	10.6	24.0	8.0	2.6
33	岡山県 (Okayama)	5.6	9.8	24.0	22.4	55.4	49.0	50.4	34.2	39.2	19.2	19.4	21.2	48.0	47.2	25.8	3.6	8.0	14.6	13.6	4.8
34	広島県 (Hiroshima)	1.8	4.4	10.3	9.9	9.5	10.6	10.7	17.6	15.6	14.7	10.4	8.9	19.5	19.9	6.2	4.6	14.3	22.2	8.9	4.4
35	山口県 (Yamaguchi)	2.9	3.5	5.4	6.8	19.4	30.8	52.6	19.3	11.1	15.9	28.4	20.1	36.1	42.3	10.8	10.5	15.6	43.7	16.9	5.2
36	徳島県 (Tokushima)	1.3	3.8	2.8	1.3	0.7	1.4	0.3	1.5	0.4	0.6	1.2	8.6	17.6	11.0	3.4	4.3	6.1	8.1	2.2	3.7
37	香川県 (Kagawa)	1.6	1.2	4.8	2.0	4.6	1.0	0.0	0.0	0.4	0.2	1.8	6.4	5.6	11.2	8.4	14.0	41.6	52.2	25.4	7.2
38	愛媛県 (Ehime)	2.0	12.4	25.2	21.0	10.7	14.2	23.7	54.8	19.5	23.5	45.3	49.2	53.2	36.7	13.5	3.2	7.5	34.7	18.2	24.2
39	高知県 (Kochi)	1.0	3.1	24.8	15.4	14.6	18.0	12.9	15.4	9.1	9.3	9.1	15.3	21.3	45.6	29.1	28.6	38.9	40.5	11.5	12.9
40	福岡県 (Fukuoka)	0.0	1.5	9.3	5.4	2.9	6.8	4.4	11.7	3.9	3.7	3.9	12.0	16.3	8.7	7.1	6.1	35.9	102.6	28.3	7.5
41	佐賀県 (Saga)	2.5	0.8	10.3	6.3	10.2	10.5	15.0	30.5	27.7	22.5	16.2	50.0	44.5	34.8	35.0	10.0	31.5	32.2	7.5	6.9
42	長崎県 (Nagasaki)	0.1	0.5	0.1	8.7	16.4	3.8	6.3	16.7	4.6	3.8	6.0	10.4	36.8	30.6	14.2	19.3	22.7	38.3	11.3	7.7
43	熊本県 (Kumamoto)	4.4	4.5	7.3	10.3	6.3	4.7	5.6	7.1	2.7	2.0	3.9	4.3	9.9	12.7	7.0	5.1	14.5	27.1	7.5	2.3
44	大分県 (Oita)	1.6	2.6	9.2	3.9	3.0	2.2	1.5	3.1	3.2	3.1	2.9	7.8	15.3	25.2	23.1	15.9	11.5	32.1	11.1	6.4
45	宮崎県 (Miyazaki)	6.1	9.3	11.0	11.4	9.8	7.3	4.7	8.8	10.2	4.0	4.4	0.7	11.5	13.6	3.6	0.4	5.1	43.7	4.3	0.7
46	鹿児島県 (Kagoshima)	1.5	3.7	6.0	6.1	3.1	3.1	5.0	10.0	12.7	16.6	11.2	6.8	25.8	30.5	10.3	0.8	7.0	21.0	3.0	1.5
47	沖縄県 (Okinawa)	11.0	15.7	6.7	9.6	6.3	2.4	9.9	64.0	146.7	135.3	116.9	89.0	128.4	106.6	46.3	24.7	28.6	69.4	22.0	6.5
		1999	2000	2001	2002	2003	2004	2005	2006	2007	2008	2009	2010	2011	2012	2013	2014	2015	2016	2017	2018

Annual surveillance data (1999-2018) of *Mycoplasma pneumoniae* pneumonia by prefecture of Japan (patient number per sentinel per year).

C

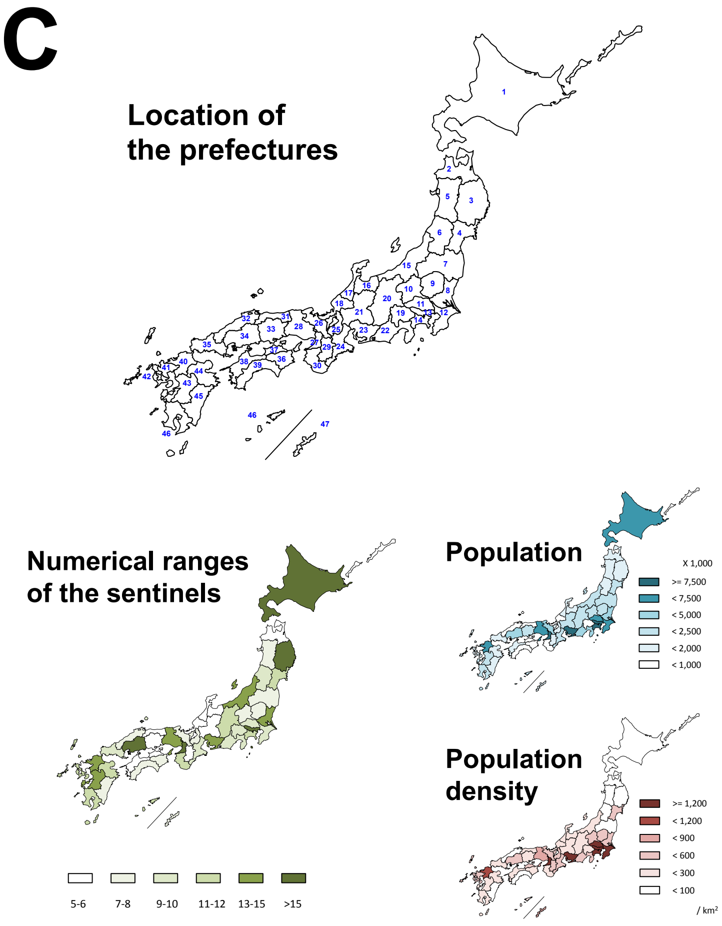

D

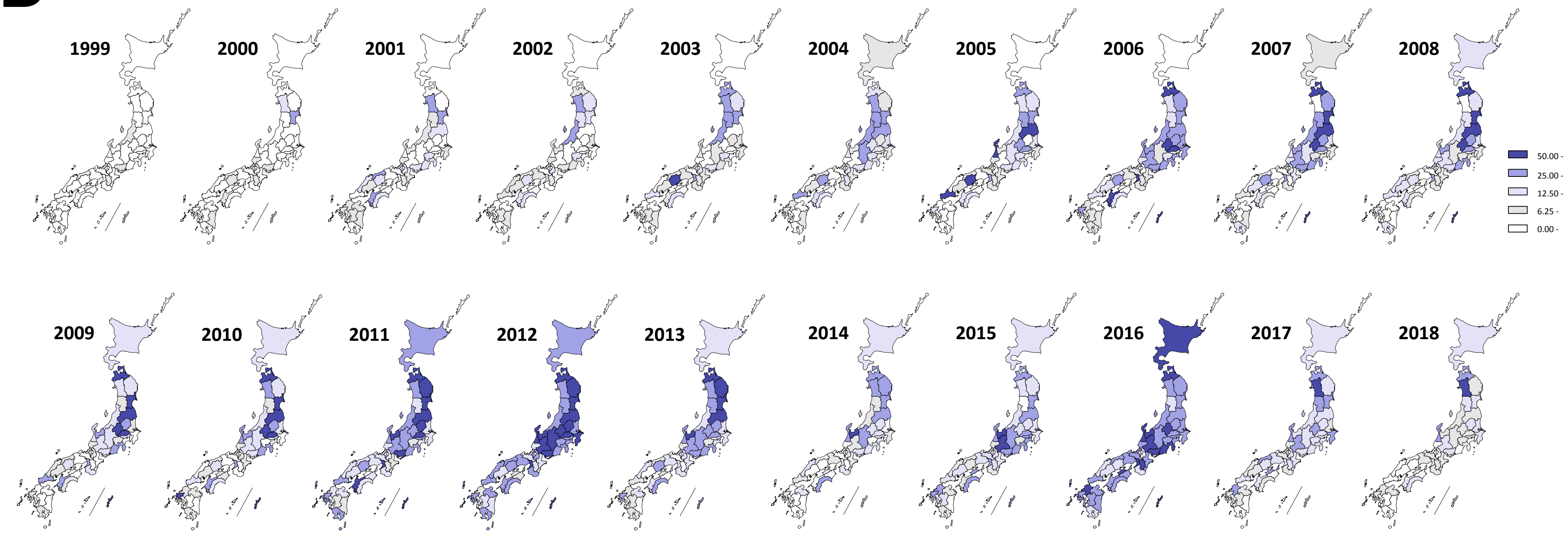

Graphical presentation of the annual surveillance data of *Mycoplasma pneumoniae* pneumonia on the map of Japan (1999-2018).

Supplementary Figure 3

Summary of *M. pneumoniae* pneumonia surveillance by the National Epidemiological Surveillance of Infectious Diseases (NESID) in Japan. **(A)** Weekly cases of *M. pneumoniae* pneumonia (per sentinel) in Japan between April 1999 to the 47th week of 2019. Nationwide data and data for each prefecture separately are shown. Data for Saitama, Kanagawa, Osaka, and Okayama where from the *M. pneumoniae* strains collected in this study are marked by rad squares. **(B)** Annual cases of *M. pneumoniae* pneumonia (per sentinel) shown by prefecture from 1999 (April) to 2018. **(C)** Location of the prefectures, numerical ranges of the sentinels, population, and population density are shown on the map of Japan. **(D)** Graphical presentation of the annual cases of *M. pneumoniae* pneumonia (per sentinel) on the map of Japan between April 1999 and 2018. The data are available from the website of the Infectious Diseases Surveillance Center, National Institute of Infectious Diseases (https://www.niid.go.jp/niid/ja/idwr.html).
