# Supplementary material for: Periodic Genotype Shifts in Clinically Prevalent Mycoplasma pneumoniae Strains in Japan
Source: Front Cell Infect Microbiol. 2020 Aug 6;10:385. doi: 10.3389/fcimb.2020.00385 (PMC7424021; doi:10.3389/fcimb.2020.00385)
Supplement: Supplementary file 1 [file Data_Sheet_1.zip › Figure S4.pdf]

A

Data source			
	**1976-1994** Sasaki et al., 1996 (1)	**1995-2005** Kenri et al., 2008 (2)	**This study** (**2011-2017** Katsukawa et al., 2019 (3))

Year

p1 types	1976	1977	1978	1979	1980	1981	1982	1983	1984	1985	1986	1987	1988	1989	1990	1991	1992	1993	1994	1995	1996	1997
**1**	3			1	1			1	7	18	4	12	2	19	7	18	13	4	1			
**1b**																						
**2**	1			3	19			1	13	2		1		1		1	7	5	12	7	13	8
**2a**																1		1				
**2b**																						
**2c**																						
**2f**																						
**2g**																						
**Total**	4	0	0	4	20	0	0	2	20	20	4	13	2	20	7	20	20	10	13	7	13	8
**Type 1 lineage**	3	0	0	1	1	0	0	1	7	18	4	12	2	19	7	18	13	4	1	0	0	0
**Type 2 lineage**	1	0	0	3	19	0	0	1	13	2	0	1	0	1	0	2	7	6	12	7	13	8
**Total**	4	0	0	4	20	0	0	2	20	20	4	13	2	20	7	20	20	10	13	7	13	8
**Type 1 lineage %**	75			25	5			50	35	90	100	92.3	100	95.0	100	90	65	40	7.69	0	0	0
**p value (Chi-squared test)**		NA	NA		<.01	NA	NA		<.1	<.01		<.01		<.01	<.05	<.01			<.01	<.01	<.01	<.01
**Dominant lineage (statistical)**					2				2	1		1		1	1	1			2	2	2	2
**Type 1 lineage MR**																						
**Type 2 lineage MR**																						
**Total MR**																						
**Type 1 lineage MR %**																						
**Type 2 lineage MR %**																						
**Total MR %**																						

p1 types	1998	1999	2000	2001	2002	2003	2004	2005	2006	2007	2008	2009	2010	2011	2012	2013	2014	2015	2016	2017	2018	2019	Total
**1**				14	26	26	28	22	14	1	20	19	13	90	59	13	15	101	167	26	2	6	**773**
**1b**																			5				**5**
**2**	9	1	24	19	22	7							2					53	68	18	27	6	**350**
**2a**	1			1		5	9	2						8					1				**29**
**2b**													1	1	1								**3**
**2c**									3			1	4	10	2	6	9	60	102	24	10	3	**234**
**2f**																			1				**1**
**2g**																			1				**1**
**Total**	10	1	24	34	48	38	37	24	17	1	20	20	20	109	62	19	24	214	345	68	39	15	**1396**
**Type 1 lineage**	0	0	0	14	26	26	28	22	14	1	20	19	13	90	59	13	15	101	172	26	2	6	**778**
**Type 2 lineage**	10	1	24	20	22	12	9	2	3	0	0	1	7	19	3	6	9	113	173	42	37	9	**618**
**Total**	10	1	24	34	48	38	37	24	17	1	20	20	20	109	62	19	24	214	345	68	39	15	**1396**
**Type 1 lineage %**	0	0	0	41.2	54.2	68.4	75.7	91.7	82.4	100	100	95	65	82.6	95.2	68.4	62.5	47.2	49.9	38.2	5.13	40	**55.7**
**p value (Chi-squared test)**	<.01		<.01				<.05	<.01	<.05		<.01	<.01		<.01	<.01			<.01	<.01	<.01	<.01		
**Dominant lineage (statistical)**	2		2				1	1	1		1	1		1	1			2	2	2	2		
**Type 1 lineage MR**									0	0	13	8	11	77	56	13	15	85	164	24	1	6	473
**Type 2 lineage MR**									0	0	0	0	0	1	0	0	0	8	3	2	0	0	14
**Total MR**									0	0	13	8	11	78	56	13	15	93	167	26	1	6	487
**Type 1 lineage MR %**									0	0	65	42.1	84.6	85.6	94.9	100	100	84.2	95.3	92.3	50	100	
**Type 2 lineage MR %**									0			0	0	5.26	0	0	0	7.08	1.73	4.76	0	0	
**Total MR %**									0	0	65	40	55	71.6	90.3	68.4	62.5	43.5	48.4	38.2	2.56	40	

B

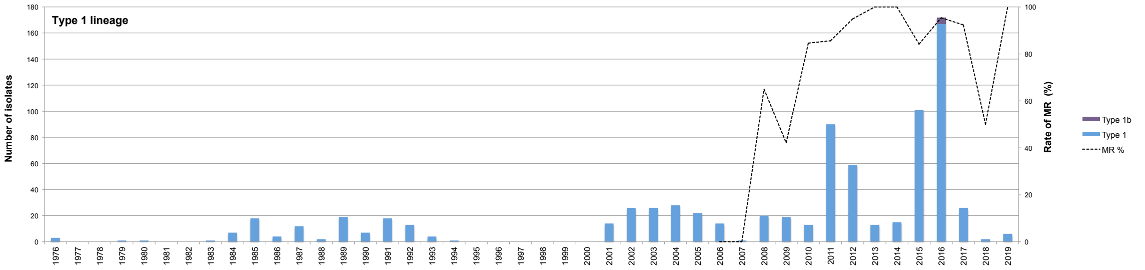

C

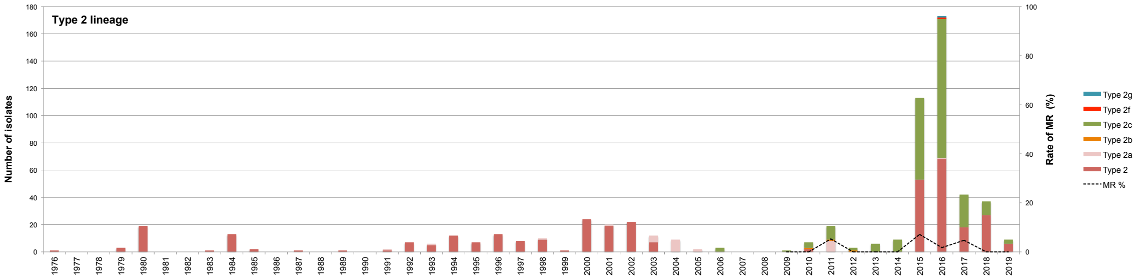

D

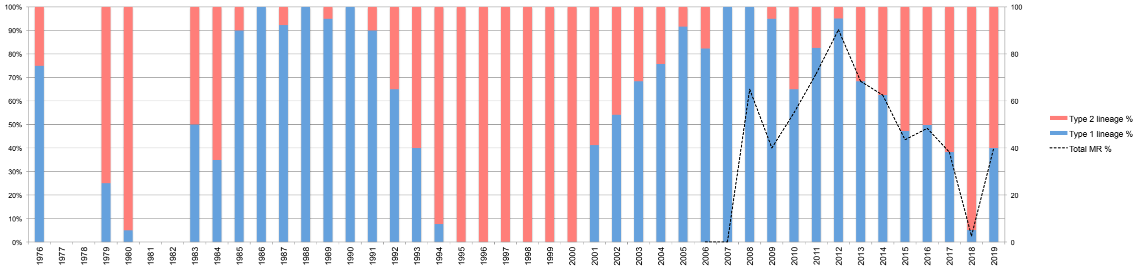

Supplementary Figure S4

Integrated data of genotyping studies of *M. pneumoniae* in Japan. Data from previous reports, Sasaki et al., 1996 (1) ; Kenri et al., 2008 (2) ; Katsukawa et al., 2019 (3), and the data from this study were integrated. **(A)** Annual numbers of *M. pneumoniae* detection, *p1* typing results, and MR detection. MR was analyzed since 2006. **(B)** Annual detection numbers and MR rate of type 1 lineage *M. pneumoniae*. **(C)** Annual detection numbers and MR rate of type 2 lineage *M. pneumoniae*. **(D)** Ratio of type 1 and 2 lineage strains and total MR rate.

1: Sasaki T, Kenri T, Okazaki N, Iseki M, Yamashita R, Shintani M, Sasaki Y, Yayoshi M. (1996).
Epidemiological study of *Mycoplasma pneumoniae* infections in Japan based on PCR-restriction fragment length polymorphism of the P1 cytadhesin gene.
J Clin Microbiol. 1996, 34,447-9.

2: Kenri T, Okazaki N, Yamazaki T, Narita M, Izumikawa K, Matsuoka M, Suzuki S, Horino A, Sasaki T. (2008).
Genotyping analysis of *Mycoplasma pneumoniae* clinical strains in Japan between 1995 and 2005: type shift phenomenon of *M. pneumoniae* clinical strains.
J Med Microbiol. 57, 469-75.

3: Katsukawa, C., Kenri, T., Shibayama, K., and Takahashi, K. (2019).
Genetic characterization of *Mycoplasma pneumoniae* isolated in Osaka between 2011 and 2017: Decreased detection rate of macrolide-resistance and increase of *p1* gene type 2 lineage strains.
PLoS One 14, e0209938.
