# Supplementary material for: Periodic Genotype Shifts in Clinically Prevalent Mycoplasma pneumoniae Strains in Japan
Source: Front Cell Infect Microbiol. 2020 Aug 6;10:385. doi: 10.3389/fcimb.2020.00385 (PMC7424021; doi:10.3389/fcimb.2020.00385)
Supplement: Supplementary file 1 [file Data_Sheet_1.zip › Figure S5.pdf]

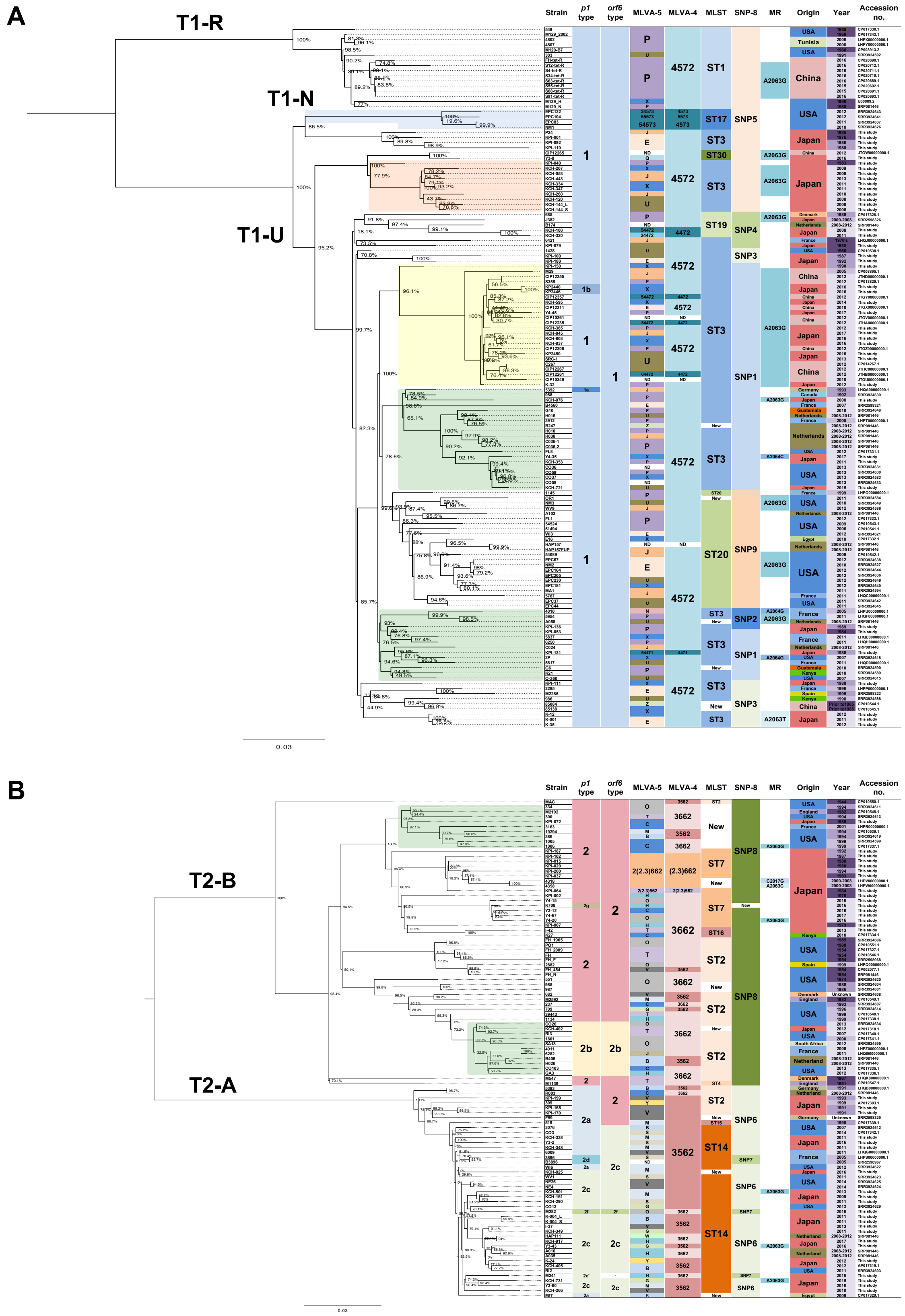

Supplementary Figure S5

Phylogenetic tree of 237 *M. pneumoniae* genomes based on whole-genome SNP analysis. *p1* , *orf6* , MLVA, MLST and SNP-8 types, MR, origin, year of isolation, and GenBank accession nos. of the strains are shown on the right of the trees.
(A) Phylogenetic tree of 136 genomes of *p1* type 1 lineage strains. **(B)** Phylogenetic tree of 101 genomes of *p1* type 2 lineage strains.
